# Completion of the Chloroplast Genomes of Five Chinese *Juglans* and Their Contribution to Chloroplast Phylogeny

**DOI:** 10.3389/fpls.2016.01955

**Published:** 2017-01-06

**Authors:** Yiheng Hu, Keith E. Woeste, Peng Zhao

**Affiliations:** ^1^Key Laboratory of Resource Biology and Biotechnology in Western China (Ministry of Education), College of Life Sciences, Northwest UniversityXi'an, China; ^2^United States Department of Agriculture Forest Service Hardwood Tree Improvement and Regeneration Center, Department of Forestry and Natural Resources, Purdue UniversityWest Lafayette, IN, USA

**Keywords:** persian walnut, ma walnut, iron walnut, chinese walnut, manchurian walnut, phylogeny, China, butternut

## Abstract

*Juglans* L. (walnuts and butternuts) is an economically and ecologically important genus in the family Juglandaceae. All *Juglans* are important nut and timber trees. *Juglans regia* (Common walnut), *J. sigillata* (Iron walnut), *J. cathayensis* (Chinese walnut), *J. hopeiensis* (Ma walnut), and *J. mandshurica* (Manchurian walnut) are native to or naturalized in China. A strongly supported phylogeny of these five species is not available due to a lack of informative molecular markers. We compared complete chloroplast genomes and determined the phylogenetic relationships among the five Chinese *Juglans* using IIumina sequencing. The plastid genomes ranged from 159,714 to 160,367 bp encoding 128 functional genes, including 88 protein-coding genes and 40 tRNA genes each. A complete map of the variability across the genomes of the five *Juglans* species was produced that included single nucleotide variants, indels (insertions and deletions), and large structural variants, as well as differences in simple sequence repeats (SSR) and repeat sequences. Molecular phylogeny strongly supported division of the five walnut species into two previously recognized sections (*Juglans/Dioscaryon* and *Cardiocaryon*) with a 100% bootstrap (BS) value using the complete cp genomes, protein coding sequences (CDS), and the introns and spacers (IGS) data. The availability of these genomes will provide genetic information for identifying species and hybrids, taxonomy, phylogeny, and evolution in *Juglans*, and also provide insight into utilization of *Juglans* plants.

## Introduction

The estimate of phylogenetic relationships plays a key role in understanding evolution and has been an essential component of evolutionary biology. In plants, much effort in reconstructing the Tree of Life has focused on the relationships of major clades, and significant advances have been made above the order or family levels (The Angiosperm Phylogeny Group III, 2009; Soltis et al., 2011). Until recently, progress in inferring phylogenetic relationships at lower taxonomic levels and among recently diverged species has been less encouraging, especially for species-rich, morphologically diverse lineages (Waterway et al., 2009). In the past few years, however, important advances have been made in multispecies coalescent approaches for resolving genome-level relationships among closely related species using next generation sequencing to resolve incomplete lineage sorting and inter-lineage hybridization (Huang et al., 2014; Carbonell-Caballero et al., 2015; Daniell et al., 2016).

Walnuts and butternuts (*Juglans*) are known for their edible nuts and high-quality wood (Manning, 1978; Aradhya et al., 2007). The genus *Juglans* includes about 21 species distributed in Asia, southern Europe, North America, Central America, western South America, and the West Indies (Manning, 1978; Stanford et al., 2000; Aradhya et al., 2007). Species of *Juglans* are diploid, with a karyotype of 2n = 2x = 32 (Woodworth, 1930; Komanich, 1982). *J. regia* (common walnut), *J. sigillata* (iron walnut), *J. cathayensis* (Chinese walnut), *J. hopeiensis* (Ma walnut), and *J. mandshurica* (Manchurian walnut) grow in China (Manning, 1978; Fjellstrom and Parfitt, 1995; Aradhya et al., 2007). *Juglans* is taxonomically and phylogenetically challenging. Classical taxonomy divides the genus into four sections (sect. *Dioscaryon*, sect. *Cardiocaryon*, sect. *Trachycaryon*, and sect. *Rhysocaryon*) mainly based on species' geographical distribution, leaf, flower, and fruit morphology (Dode, 1909; Manning, 1978). Molecular evidence, however, including sequence data from the internal transcribed spacer (ITS), five chloroplast DNA spacer sequences (*atpB-rbcL, psbA-trnH, trnS-trnfM, trnT-trnF*, and *trnV-16S rRNA*), a hyper-variable *matK*, and restriction fragment length polymorphisms (RFLPs), has been interpreted as supporting three or four sections (Fjellstrom and Parfitt, 1995; Stanford et al., 2000; Aradhya et al., 2007).

Chinese *Juglans* species are divided into two sections (sect. *Dioscaryon* and sect. *Cardiocaryon*). Common walnut (*J. regia*) and Iron walnut (*J. sigillata*) belong to sect. *Dioscaryon*, and the other three species (*J. cathayensis, J. hopeiensis*, and *J. mandshurica*) belong to sect. *Cardiocaryon* (Dode, 1909; Fjellstrom and Parfitt, 1995; Stanford et al., 2000; Aradhya et al., 2007). Common walnut (*J. regia*) is native to the mountainous regions of central Asia (Pollegioni et al., 2015), while Iron walnut (*J. sigillata*) is indigenous to China, and distributed mainly in southwestern China (Wang et al., 2015). Chinese walnut (*J. cathayensis*) is widely distributed in southern China (Bai et al., 2014; Dang et al., 2015), while *J. mandshurica* is mainly distributed in northern China, northeast China, and the Korean Peninsula (Wang et al., 2016). *J. hopeiensis* is narrowly distributed in northern China in the hilly, mid-elevation area between Hebei province, Beijing, and Tianjin (Hu et al., 2015). A strongly supported phylogeny of these five species is not available due to a lack of informative molecular markers (Fjellstrom and Parfitt, 1995; Stanford et al., 2000; Aradhya et al., 2007). Studies of gene flow and introgression have concluded *J. regia* and *J. sigillata* are particularly closely related, and some have questioned whether they are distinct (Wang et al., 2008, 2015). Aradhya et al. (2007) used ITS, RFLP, and cpDNA sequence data to suggest *J. regia* and *J. sigillata* are distinct species. *J. cathayensis* and *J. mandshurica* were combined into one species in Flora of China (English version) (Lu et al., 1999), which does not consider *J. hopeinesis* (Kuang and Lu, 1979; Aradhya et al., 2004, 2007) a valid taxon. In addition, some previous phylogenetic studies of *Juglans* omitted *J. hopeiensis* and *J. sigillata* (Fjellstrom and Parfitt, 1995; Stanford et al., 2000; Aradhya et al., 2007). Thus, the phylogeny and systematics of the five Chinese walnut (*Juglans*) species is uncertain.

In this study, we combined *de novo* and reference-guided assembly of five Chinese walnut (*Juglans*) species' whole chloroplast genomes (Cpgs). This is the first comprehensive Cpg analysis of multiple *Juglans* species. Our aims were: (1) to investigate global structural patterns of whole chloroplast genome of five *Juglans* species including genome structure, gene order, and gene content; (2) to examine variations of simple sequence repeats (SSRs) and large repeat sequence in the whole Cpgs of *Juglans*; (3) to identify divergence hotspots as regions potentially under selection pressure; and (4) to construct a chloroplast phylogeny for the five Chinese *Juglans* species using their whole cp DNA sequences, protein coding sequences, and the introns and spacers.

## Materials and methods

### Taxon sampling, plant material, and deposition of voucher

Fresh leaves of four *Juglans* species were collected from different mountains in China, including a *J. mandshurica* tree growing in the Xiaolongmen National Forest Park, a *J. sigillata* tree from Lijiang, Yunan, a *J. hopeiensis* tree growing Laishui, Beijing, and a *J. cathayensis* tree growing in the Qingling Mountains (Table 1). The leaves were dried in silica gel and stored at −4°C. The leaves of *J. regia* were collected fresh from a tree growing the orchard of Northwest University, Shaanxi, China. Voucher specimens of each of the sampled trees were deposited at the herbarium of Northwest University, Xi'an, China. All the DNA samples were stored at Evolutionary Botany Lab, Northwest University, Xi'an, China. High-quality genomic DNA was extracted using a modified CTAB method (Zhao and Woeste, 2011). The DNA concentration was quantified using a NanoDrop spectrophotometer (Thermo Scientific, Carlsbad, CA, USA). The final DNA concentration >30 ng μL^−1^ were chosen for further Illumina sequencing. We sequenced the complete chloroplast genome of *J. regia* with the Illumina MiSeq sequencing platform (Sangon Biotech, Shanghai, China). We assembled the chloroplast genomes using SPAdes v3.6.2 (Bankevich et al., 2012) (http://bioinf.spbau.ru/spades) and annotated them with CpGAVAS (http://www.biomedcentral.com/1471-2164/13/715) (Liu et al., 2012a; Hu et al., 2016). We sequenced the complete Cpg of four *Juglans* species using Illumina HiSeq 2500 sequencing technology via a combination of *de novo* and reference-guided assembly based on the Cpg of *J. regia* (Hu et al., 2016, NCBI Accession number: KT963008). A paired-end (PE) library with 350-bp insert size was constructed using the Illumina PE DNA library kit according to the manufacturer's instructions and sequenced using an Illumina Hiseq2500 by Novogene (http://www.novogene.com, China).

**Table 1:** **Summary statistics for assembly of five ***Juglans*** species chloroplast genomes**.

**Genome features**	***Juglans regia***	***Juglans sigillata***	***Juglans hopeiensis***	***Juglans cathayensis***	***Juglans mandshurica***
Size (bp)	160367	160350	159714	159730	159729
LSC length (bp)	89872	89872	89316	89333	89331
SSC length (bp)	18423	18406	18352	18351	18352
IR length (bp)	26036	26036	26023	26023	26023
Coding (bp)	80475	80475	80202	80110	80344
Noncoding (bp)	79892	79875	79512	79620	79385
Number of genes	129	129	129	129	129
Protein-coding genes	88	88	88	88	88
tRNA genes	40	40	40	40	40
rRNA genes	8	8	8	8	8
Number of genes duplicated in IR (rRNA/tRNA/gene/Pseudogenes)	19 (4/7/7/1)	19 (4/7/7/1)	19 (4/7/7/1)	19 (4/7/7/1)	19 (4/7/7/1)
GC content (%)	36.1	36.1	36.1	36.1	36.1
GC content in LSC (%)	33.6	33.6	33.6	33.7	33.7
GC content in SSC (%)	29.8	29.8	29.8	29.8	29.8
GC content in IR (%)	42.6	42.6	42.6	42.5	42.5
Sequencing Platform	Illumina Miseq	Illumina HiSeq	Illumina HiSeq	Illumina HiSeq	Illumina HiSeq
Raw reads	6321912	12382845	10285876	13320133	11903351
Raw Base (G)	1.9	3.1	2.57	3.33	2.98
Average read length (bp)	300	150	150	150	150
Average insert size (bp)	350	350	350	350	350
Number of assembled reads	1846010	804634	1118104	689686	1055940
Source	Xi'an, Qinling	Lijiang, Yunnan	Laishui, Beijing	Lantian, Qinling	Xiaolongmen, Beijing

### Chloroplast genome sequencing, assembly, and gap filling

Raw reads with sequences shorter than 50 bp or with more than the allowed maximum percentage of ambiguous bases (2%) were removed from the total NGS PE reads using the NGSQC toolkit v2.3.3 (Patel and Jain, 2012) trim tool. After trimming, high-quality PE reads were assembled using MIRA v4.0.2 (Chevreux et al., 2004) assembler. Then, to further assemble the Cpg, some ambiguous regions were picked out for extension with a baiting and iteration method based on MITObim v1.8 (Hahn et al., 2013). A *de novo* assembly strategy combined with a reference-based assembly allowed us to reconstruct each Cpg. Reads were then remapped to references for each taxon to check for mis-assemblies or rearrangements using Geneious v8.0.2 (http://www.Geneious.com; Kearse et al., 2012) and reads matching the draft reference were assembled *de novo*, also in Geneious, using suggested settings. Inverted repeat boundaries were determined and verified by remapping reads in Geneious. Lastly, primers were developed with Primer3 (Untergrasser et al., 2012) to close low coverage gaps between contigs (for a few single end datasets). Small gaps in the assemblies were bridged by designing custom primers for PCR (Table S1) based on their flanking sequences, followed by conventional Sanger sequencing. The PCR primers were designed using *J. regia* sequences when they appeared identical to our original *de novo* assembly (Hu et al., 2016). Eleven primer pairs were used to validate junctions using PCR based sequencing in each of five *Juglans* Cpgs. PCR amplification was carried out on a SimpliAmp Thermal Cycler (Applied Biosystem, USA) in 20 μL reaction volumes (10 μL 2 × PCR Master Mix including 0.1 U Taq polymerase/μL; 500 μM each dNTP; 20 mM Tris-HCl (pH 8.3); 100 mM KCl; 3.0 mM MgCl_2_ (Tiangen, Beijing, China), 0.5 μL each primer, 2 μL BSA, 2 μL of 10 ng/μL DNA). The PCR was programmed for 3 min at 94°C followed by 35 cycles of 15 s at 93°C, 1 min at annealing temperature (60°C), 30 s at 72°C and extension of 10 min at 72°C. After PCR amplification, fragments were sequenced by Sangon Biotech (Shanghai, China). All newly generated sequences were deposited in GenBank (Table S1).

### Genome annotation and analysis

The completed genome sequences were imported into the online program Dual Organellar Genome Annotator (DOGMA, Wyman et al., 2004) for annotation, coupled with manual investigation of the positions of start and stop codons and boundaries between introns and exons. Putative starts, stops, and intron positions were determined by comparison with homologous genes in other chloroplast genomes using MAFFT v7.0.0 (Katoh and Standley, 2013). Genes and open reading frames (ORF) that may not have been annotated were identified with the aid of Geneious. In addition, all tRNA genes were further verified online using tRNAscan-SE search server (Lowe and Eddy, 1997) (http://lowelab.ucsc.edu/tRNAscan-SE/). The circular *Juglans regia* chloroplast genome map was drawn using Organellar Genome DRAW (Lohse et al., 2013). Genome annotation was performed in Geneious, and the GC-content of protein-coding genes, tRNA genes, introns and intergenic spacers (IGSs) was determined on the basis of their annotation. Cpg comparison among the five *Juglans* species was performed with VISTA (Frazer et al., 2004). Genome, protein coding gene, intron, and spacer sequence divergences were evaluated using DnaSP v5.10 (Librado and Rozas, 2009) after alignment. For the protein coding gene sequences, introns, and spacers, every gene or fragment was annotated using the software Geneious v8.0.2 (http://www.Geneious.com; Kearse et al., 2012). For purposes of the subsequent phylogenetic analysis and plant identification, the complete Cpg of each *Juglans* species was compared and diagramed using VISTA to show sequence divergence.

### Repeat sequencing analysis

The genomic sequences were analyzed to identify potential microsatellites (simple sequence repeats orSSRs, i.e., mono-, di-, tri-, tetra-, penta-, and hexanucleotide repeats) using MISA software (http://pgrc.ipk-gatersleben.de/misa/) with thresholds of ten repeat units for mononucleotide SSRs and five repeat units for di-, tri-, tetra-, penta-, and hexanucleotide SSRs. The web-based software REPuter (Kurtz et al., 2001) (http://bibiserv.techfak.uni-bielefeld.de/reputer/) was used to analyze the repeat sequences, which included forward, reverse, complement, palindromic and tandem repeats with minimal lengths of 30 bp and edit distances of less than 3 bp. The large repeat sequences were analyzed by using the Web-based Tandem Repeats Finder (http://tandem.bu.edu/trf/trf.html). We investigated if the repeated elements identified in the chloroplast of *J. regia* were also present in other four other Chinese *Juglans* species by aligning their cp genomes using Geneious v8.0.2 (http://www.Geneious.com; Kearse et al., 2012). Tandem repeat sequences (>10 bp in length) were detected using the online program Tandem Repeats Finder (Benson, 1999), with 2, 7, and 7 set for the alignment parameters match, mismatch, and indel, respectively. The minimum alignments core and maximum period size were 80 and 500, respectively.

### Mutation events analysis, substitution rate analyses, and inference of rate changes

To identify the microstructural mutations of *Juglans*, the five aligned sequences were further analyzed using DnaSP v5 (Librado and Rozas, 2009) and MEGA v5.0 (Tamura et al., 2011). Indel and SNP events were counted and positioned in the cp genome using DnaSP v5. Signatures of natural selection were studied for every chloroplast gene located outside of the inverted repeats region. Selective pressures (*K*_*A*_/*K*_*S*_) were computed with the codeml tool from PAML package v4.0 (Yang, 2007) using a YN00 model to test every gene sequence. We used the KaKs_calculator program to check the selective pressures (*K*_*A*_/*K*_*S*_) using same model as YN (Zhang et al., 2006). To avoid potential convergence biases, those genes with few mutations were filtered out from selective pressure analysis.

### Phylogenetic analysis

The *Juglans* Cpg sequences from the finalized data set were aligned with MAFFT v7.0.0 (Katoh and Standley, 2013). The analyses were carried out based on the following three data sets: (1) the complete cp DNA sequences; (2) protein coding sequences; (3) the introns and spacers. We conducted ML analyses using each of the data sets separately. The phylogenetic analyses were carried out using the Cpgs of all five *Juglans* species plus eight other species with complete Cpgs (Table S2). The Maximum Likelihood (ML) phylogenetic tree analysis was conducted using RAxML v8.0 (Stamatakis, 2014) under GTRGAMMA model. For ML analysis, difference general time reversible models were performed with all three data sets. For all analyses, 10 independent ML searches were conducted, bootstrap support was estimated with 1000 bootstrap replicates, and bootstrap proportions were drawn on the tree with highest likelihood score from the 10 independent searches. The choice of substitution model for each partition was primarily determined by using Modeltest v3.7 (Posada and Crandall, 1998) with the Akaike information criterion (AIC) (Posada and Buckley, 2004). Maximum Parsimony (MP) phylogenetic analyses were performed in MEGA v5.0 (Tamura et al., 2011) using 1000 bootstrap replicates.BI trees were produced by MrBayes v3.2.6 (Huelsenbeck and Ronquist, 2001; Ronquist and Huelsenbeck, 2003; Altekar et al., 2004) with the setting of 1,000,000 generations and stopval = 0.01, under GTRGAMMA model with one cold and three incrementally heated Markov Chain Monte Carlo (MCMC) run simultaneously (Ronquist and Huelsenbeck, 2003) in two parallel runs sampling every 1000 generations. The first 25% of the trees were discarded as burn-in. The remaining trees were used for generating the consensus tree. The phylogenetic relationships and divergence time between lineages were estimated using Bayesian inference method BEAST v1.8.0 (Drummond et al., 2012). Calibration of the Juglandaceae and Fagaceae split (73.4 ± 0.1 Myr) was based on references in Thomas et al. (2012) and Hedges et al. (2015). The GTRAGMMA nucleotide substitution model was selected using software MODELTEST v3.7 (Posada and Crandall, 1998). A relaxed clock with lognormal distribution of uncorrelated rate variation was specified. A normal prior probability distribution was used to accommodate the uncertainly of prior knowledge. Two independent Markov chains of 10,000,000 generations, sampled every 10,000 th iteration, were generated. An adequate effective sample size (larger than 200) and convergence of the Markov chain Monte Carlo chains were diagnosed in Tracer v1.6 with the first 10% samples discarded as burn-in (Drummond et al., 2012). The phylogenetic trees were then complied into a maximum clade credibility tree using TreeAnnotator v1.8.0 (Drummond et al., 2012) and the program FigTree v1.3.1 (Drummond et al., 2012) to visualize mean node ages and highest posterior density (HPD) intervals at 95% (upper and lower) for each node and to estimate branch lengths and divergence times.

## Results

### Genome assembly and PCR-based gap filling

Using the Illumina HiSeq system, five *Juglans* species were sequenced to produce a total of 10,285,876 to 13,320,133 bp paired-end raw reads from four *Juglans* species, while Common walnut (*J. regia*) had 6,321,912 bp raw reads (Table 1). After aligning the paired-end reads with the reference Cpg (common walnut, *J. regia*), 689,686 to 1,118,104 bp Cpg reads were assembled (Table 1). The four Chinese *Juglans* Cpgs were deposited in NCBI GenBank (accession numbers, KX671976, KX671977, KX671975, and KT963008).

### General features of the five chinese walnut (*Juglans*) chloroplast genomes

**Figure 1:**
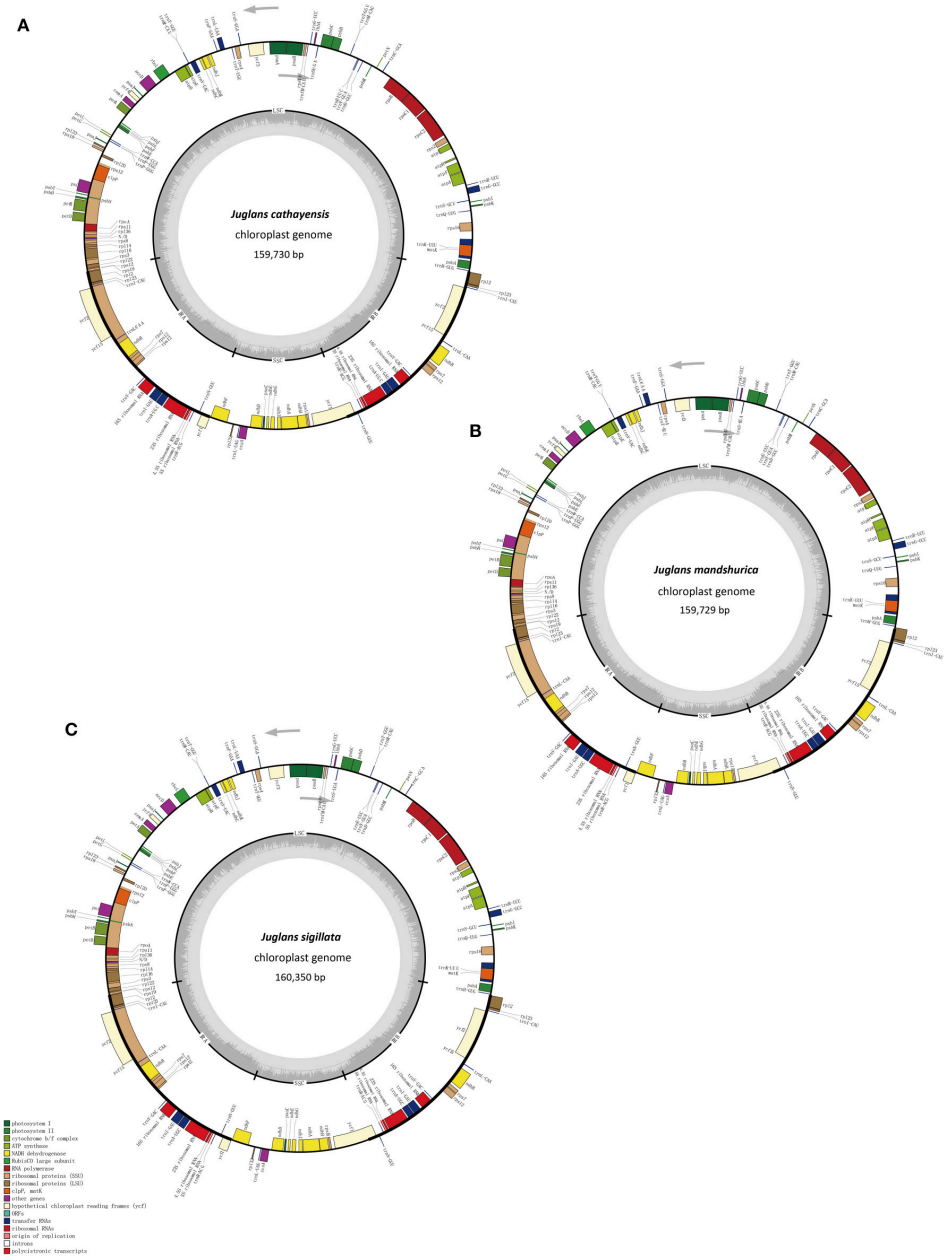
**Chloroplast genome maps of three ***Juglans*** species. (A)** *J. cathayensis* chloroplast genome, **(B)** *J. mandshurica* chloroplast genome, **(C)** *J. sigillata* chloroplast genome. Genes drawn outside the outer circle are transcribed clockwise, and those inside are transcribed counter-clockwise. Genes belonging to different functional groups are colorcoded. Thedark gray in the innercircle indicates GC content of the chloroplast genomes.

The five *Juglans* Cpgs ranged from 159,714 bp (*J. hopeiensis*) to 160,367 bp (*J. regia*), the average Cpg sequence length was 159,978 bp (Figure 1, Table 1). The coding sequence of the five *Juglans* Cpg ranged from 80,110 bp (*J. cathayensis*) to 80,475 bp (*J. regia* and *J. sigillata*), while the LSC length and SSC length ranged from 89,316 bp (*J. hopeiensis*) to 89, 872 bp (*J. regia* and *J. sigillata*) and 18,351 bp (*J. cathayensis*) to 18, 423 bp (*J. regia*), respectively (Table 1). For all five Cpgs the average GC content was 36.1% (Table 1). There are four introns located in the IR region and 13 introns in the LSC region in each of the Cpgs. There was only one gene (*ndhA*) located in SSC region (Table 2). All five Cpgs included a large single-copy (LSC) region of 89,316 to 89,872 bp, a small single-copy (SSC) region of 18,351 to 18,406 bp, and the inverted repeats (IR)were 26,023 bp (Figure S1, Table 1). All five walnut Cpgs encoded 128 functional genes, including 88 protein-coding genes, 40 tRNA genes, and 8 ribosomal RNA genes (Table 1). There were 18 intron-containing genes (one class I intron in *trn-UAA* and 17 class II introns), of which three genes *rps12, clpP*, and *ycf3*, contained two introns and the rest had only one intron each (Table 2).In addition, there were two pseudogenes: *infA* and *ycf15*, in which several internal stop codons were identified. The *ycf15* gene displayed exactly the same structure in all five Chinese *Juglans* Cpgs. The pseudogene *infA* contained internal stop codons which differed among the five *Juglans* Cpg.

**Table 2:** **Gene contents in five ***Juglans*** species chloroplast genomes**.

**Category of genes**	**Group of gene**	**Name of gene**				
Self-replication	Ribosomal RNA genes	*rrn4.5^a^*	rrna5^a^	*rrn16^a^*	*rrn23^a^*	
	Transfer RNA genes	*trnA-*UGC^a^^b^	*trnC*-GCA	*trnD*-GUC	*trnE*-UUC	*trnF*-GAA
		*trnfM*-CAU	*trnG*-GCC^b^	*trnG*-UCC	*trnH*-GUG	*trnI*-CAU^a^
		*trnI*-GAU^a^^b^	trnK-UUU^b^	*trnL*-CAA^a^	*trnL*-UAA^b^	*trnL*-UAG
		*trnM*-CAU^a^	*trnN*-GUU^a^	*trnP*-GGG	*trnP*-UGG	*trnQ*-UUG
		*trnR*-ACG^a^	*trnR*-UCU	*trnS*-GCU	*trnS*-GGA	*trnS*-UGA
		*trnT*-GGU^a^	*trnT*-UGU	*trnV*-GAC^a^	*trnV*-UAC^a^	*trnW*-CCA
		*trnY*-GUA				
	Small subunit of ribosome	*rps2*	*rps3*	*rps4*	*rps7*^a^	*rps8*
		*rps11*	*rps12*^a^^c^	*rps14*	*rps15*	*rps16*^b^
		*rps18*	*rps19*			
	Large subunit of ribosome	*rpl2*^a^^b^	*rpl14*	*rpl16*^b^	*rpl20*	*rpl22*
		*rpl23*^a^	*rpl32*	*rpl33*	*rpl36*	
	DNA-dependent RNA polymerase	*rpoA*	*rpoB*	*rpoC1*^b^	*rpoC2*	
	Tanskational initiation factor	*infA*^d^				
Genes for photosynthesis	Subunits of NADH-dehydrogenase	*ndhA*^b^	*ndhB*^a^^b^	*ndhC*	*ndhD*	*ndhE*
		*ndhF*	*ndhG*	*ndhH*	*ndhI*	*ndhJ*
		*ndhK*				
	Subunits of photosystem I	*psaA*	*psaB*	*psaC*	*psaI*	*psaJ*
		*ycf3*^c^	*ycf4*			
	Subunits of photosystem II	*psbA*	*psbC*	*psbD*	*psbE*	*psbF*
		*psbH*	*psbI*	*psbJ*	*psbK*	*psbL*
		*psbM*	*psbN*	*psbT*		
	Subnuits of cytochrome b/f complex	*petA*	*petBb*	*petD*^b^	*petG*	*petL*
		*petN*				
	Subunits of ATP synthase	*atpA*	*atpB*	*atpE*	*atpF*^b^	*atpH*
		*atpI*				
	Subunits of rubisco	*rbcL*				
Other genes	Maturase	*matK*				
	Protease	*clpP*^c^				
	Envelope membrane protein	*cemA*				
	Subunit of Acetyl-CoA-carboxylase	*accD*				
	C-type cytochrome synthesis gene	*ccsA*				
Genes of unknown function	Conserved open reading frames	*ycf1*^a^	*ycf2*^a^	*ycf15*^a^^d^		

a *Two gene copies in IRs*.

b *Gene containing a single intron*.

c *Gene containing two introns*.

d *Pseudogene*.

### Conservation within *Juglans* Cps and comparison with Fagaceae and Betulaceae

**Figure 2:**
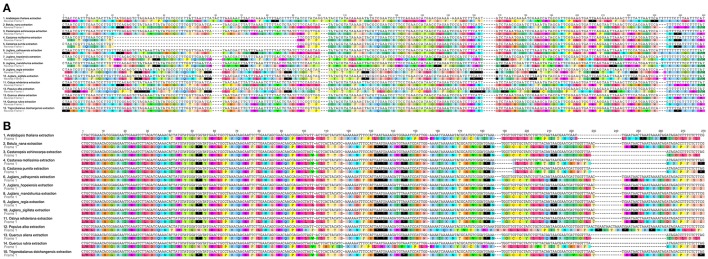
**Alignment of two pseudogenes in the five Chinese ***Juglans*** species and 10 eudicot outgroups chloroplast genome**. **(A)** *infA*. **(B)** *ycf15*. The black box with an asterisk represents stop codons.

When duplicated genes in IR regions were counted only once, all five *Juglans* Cpgs harbored 128 functional genes (except eight rRNA and pseudogenes *ycf15* and *infA*) arranged in the same order, including 88 protein-coding genes and 40 tRNAs (Table 2). Fourteen of the protein-coding genes and six of the tRNA genes contained introns, 19 of which contained a single intron, whereas four had two introns (Table 2). The numbers of protein-coding genes in the Cpgs of the five Chinese *Juglans* was similar to the number of protein-coding genes in the Betulaceae and Fagaceae, two closely related plant families. As described above, *ycf15* was a pseudogene in all five Chinese *Juglans*; it is also non-functional in the Betulaceae, and Fagaceae except in *Q. rubra*. We identified seven internal stop codons in the *ycf15* sequence of Chinese *Juglans* (Figure 2B). The *infA* gene was also present as a pseudogene in all five Chinese *Juglans* Cpgs because of several stop codons. By contrast, *infA* appears to be a protein-coding gene in *Quercus, Castanopsis*, and *Trigonobalanus*. In *Castanea*, the *infA* gene contains a long indel (70 bp) rather than an internal stop codon (Figure 2). In this study, we identified nine internal stop codons in the *infA* sequence of *J. regia* and *J. sigillata* (sect. *Dioscaryon*). By contrast, we found five, five, and two internal stop codons in the *infA* sequence of *J. hopeiensis, J. mandshurica*, and *J. cathayensis*, respectively (Figure 2A).

All five *Juglans* Cpg IR regions were well conserved, including gene number and gene order, but they exhibited obvious differences at the single-copy (SC) boundary regions (Figure S1). The nucleotide sequence length of SSC regions ranged from 18,351 to 18,423 bp (72 bp difference), while the nucleotide sequence length of the IR regions ranged from 26,023 to 26,036 bp (13 bp difference) (Table 1). The nucleotide sequence differences were mainly found between members of the two sections (sect. *Dioscaryon*, and sect. *Cardiocaryon*). Within the IR region, the gene *ycf2* had two SNPs, and *ycf7* had one SNP. There were two polymorphisms (12 bp indel and 6 bp indel) in the *ycf2*-trnV-GAC spacer region, and one SNP in the *rRNA*-*trnI*-*GAU* 16S interval, one SNP in the intron of *trnI*-*GAU*, six in the *rRNA* 23S, and one in *rRNA*-*trnR*-*ACG*. The *trnR*-*ACG*-*trnN*-*GUU* spacer region had three SNPs. The gene *ycf1* had six SNPs and one indel of 7 bp (Table S3). The gene *ycf1* crossed into the SSC region, and the pseudogene fragment *ycf1* was located in the IRA region at 1158 to 1162 bp.

**Figure 3:**
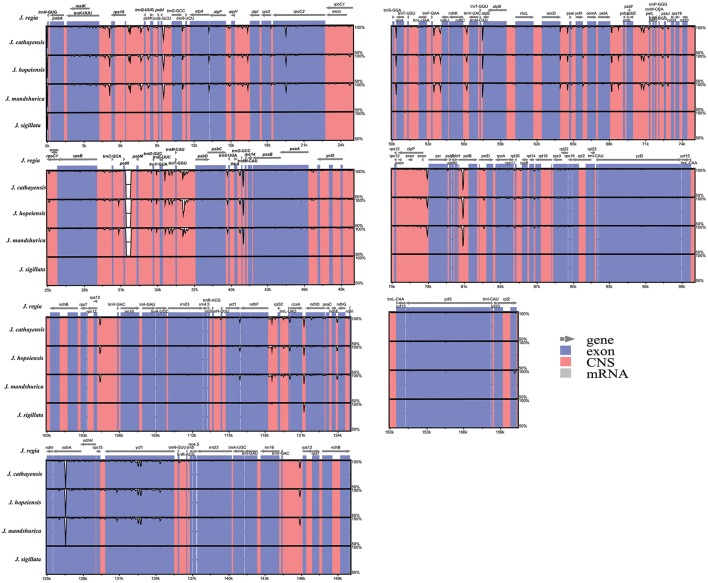
**Sequence identity plot comparing the five ***Juglans*** chloroplast genomes with ***J. regia*** as a reference by using mVISTA**. Vertical scale indicates the percentage of identity ranging from 50 to 100%. Coding regions are marked in blue and non-coding regions are marked in red. Gray arrows indicate the position and direction of each gene.

The coding regions of the Cpgs were more highly conserved than the non-coding regions, as expected (Figure 3), but there were differences among the five species. The most dissimilar coding regions were *ndhA* and *rpoC2* (Figure 3). Other evolutionary differences among the five cp genomes were inferred from differences in genome size in general and, in particular, differences in the size of the single copy (SC) region (Figure S1).

### Microsatellite polymorphims and repeat sequences

**Figure 4:**
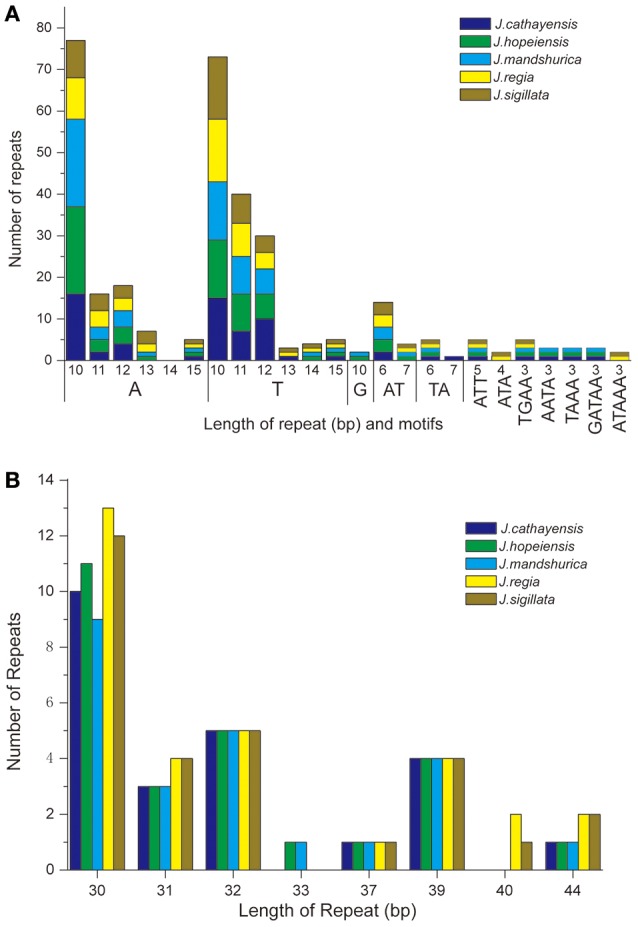
**Analysis of repeated sequences in the five Chinese ***Juglans*** chloroplast genomes. (A)** Frequency of selected motifs of simple sequence repeats (SSRs) >10 bp. **(B)** Frequency of repeat sequences of length >40 bp.

Each *Juglans* Cpg contained 66 to 83 SSRs at least 10 bp in length (Table 3, Figure 4A, Table S4). Among these SSRs (about 73 SSRs per Cpg), most were located in noncoding sections of the LSC/SSC region (96.3% of the total occurrences), and about 11 per Cpg were in protein-coding genes (*ycf1, rpoC1, ropC2, rpoB*, and *atpB*) (Table 3, Table S4). *J. hopeiensis* and *J. mandshurica* included about 17 more SSR loci in their Cpgs than the other three species. Mono-, di-, trin-, tetra-, penta-, and complex nucleotide SSRs were detected in every species, the mononucleotide, complex nucleotide, and dinucleotide SSRs averaged 64.8, 10.4, and 5.6%, of all SSRs, respectively. SSRs in walnut Cpgs are especially rich in AT. Nearly all SSRs (84.0%) were mononucleotide A/T repeats; only one or two C/G mononucleotide SSRs per genome were present. Among dinucleotide SSRs, AT/TA repeats were the most common (typically about seven per Cpg), trinucleotide SSRs (ATT/ATA) repeats were present in a small number of loci (one or three, depending on species), and depending on species, from 8 to 11 loci contained complex nucleotide repeats (Table 3, Figure S2, Table S4). AAAAT/ATTTT SSRs and AAATAT/ATATTT SSRs were only found in *J. regia* and *J. sigillata* (section *Dioscaryon*), and AAGAT/ATCTT repeat units were only found in *J. cathayensis, J. hopeiensis* and *J. mandshurica*) (Table 3, Figure S2, Table S4).

**Table 3:** **Summary of the simple sequence repeats (SSRs) in five ***Juglans*** species**.

**Species**	**SSR Loci (N)**	**P1 Loci^a^ (N)**	**P2 Loci (N)**	**P3 Loci (N)**	**P4 Loci (N)**	**P5 Loci (N)**	**Pc Loci (N)**	**LSC**	**SSC**	**IRa**	**IRb**
*J.cathayensis*	66	57	4	1	3	1	/	53	9	2	2
*J.hopeiensis*	83	62	5	1	3	1	11	67	12	2	2
*J.mandshurica*	83	62	5	1	3	1	11	67	12	2	2
*J.regia*	67	48	5	2	1	1	8	57	8	1	1
*J.sigillata*	66	49	5	2	1	1	8	56	8	1	1

a *P1 to P5indicate SSR loci with mono-, di-, tri-, tetra-, and pentanucleotide repeats, respectively.Pc indicates complex nucleotide repeats*.

### Long repeat analysis

*Juglans* Cpgs contained numerous forward repeats, palindromic repeats, and reverse repeats of at least 30 bp with a sequence identity ≥ 90% (Figure 4B, Table S5). These “long repeats” ranged from 30 to 44 bp in length and were repeated twice. Protein-coding genes (e.g., *rpoC1, psaB, petB*, and *ycf2*) contained a range of five to seven long repeat sequences (across species). Species also varied somewhat for number of long repeat sequences located in the intergenic regions (*J. regia n* = 24; *J. sigillata n* = 22; *J. hopeiensis n* = 21; *J. mandshurica, n* = 20; *J. cathayensis n* = 19; Table S5). Depending upon species, we observed 12 or 13 forward repeats, 11 to 16 palindromic repeats, one or two reverse repeats, and one complementary repeat (only seen in *J. hopeiensis*)(Table 4, Table S5). The longest forward repeat unit was 44 bp; it was located in the *psbT*-*psbN* intergenic spacer of the LSC region of *J. regia* and *J. sigillata*. A different 44 bp repeat was located in the protein-coding genes *psaB-psaA* in the LSC of *J. cathayensis, J. hopeiensis*, and *J. mandshurica* (Table S5). In the sections *Juglans/Dioscaryon, J. sigillata* and *J. regia* each contained 13 forward repeats and two reverse repeats, and 16 (*J. regia*) or 13 (*J. sigillata*) palindromic repeats (Table 4, Table S5). In the section *Cardiocaryon, J. cathayensis* contained 13 forward and 11 palindromic repeats, *J. hopeiensis* contained 13 forward, 11 palindromic, onereverse, and one complementary repeat, and *J. mandshurica* contained 12 forward, 12 palindromic, and 1 reverse repeat (Table 4, Table S5). Tandem repeats of more than 20 bp and 100% sequence identity were identified in the intergenic spacers of *trnK*-*UUU*-*rps16* (one repeat each in *J. hopeiensis, J. mandshurica*, and *J. cathayensis*); *trnE*-*UUC-trnT*-*GGU* (*J. regia*, 1; *J. sigillata*, 1; *J. hopeiensis*, 2; *J. mandshurica*, 1; *J. cathayensis*, 1); *trnT*-*GGU*-*psbD* (*J. regia*, 1; *J. sigillata*, 1; *J. hopeiensis*, 1; *J. mandshurica*, 2; *J. cathayensis*, 1); *lhbA*-*trnG*-*UCC* (*J. hopeiensis*, 1; *J. mandshurica*, 1; *J. cathayensis*, 1); *ndhC*-*trnV*-*UAC* (every *Juglans* species had one repeat); *trnF*-*GAA*-*ndhJ* (*J. regia*, 1; *J. sigillata*, 1); and *trnG*-*UCC*-*trnfM*-*CAU* (*J. regia*, 1; *J. sigillata*, 1). Two identical tandem repeats were found in the protein-coding regions of all five *Juglans* Cpgs (Table S6).

**Table 4:** **Summary of the long repeat^**a**^ sequences in five ***Juglans*** species chloroplast genomes**.

**Species**	**Forward**	**Palindromic**	**Reverse**	**Complement**
*J. cathayensis*	13	11	0	0
*J. hopeiensis*	13	11	1	1
*J. mandshurica*	12	12	1	0
*J. regia*	13	16	2	0
*J. sigillata*	13	14	2	0

a *Long repeat sequences were at least 30 bp with a sequence identity ≥90%*.

### Divergence hotspots

**Figure 5:**
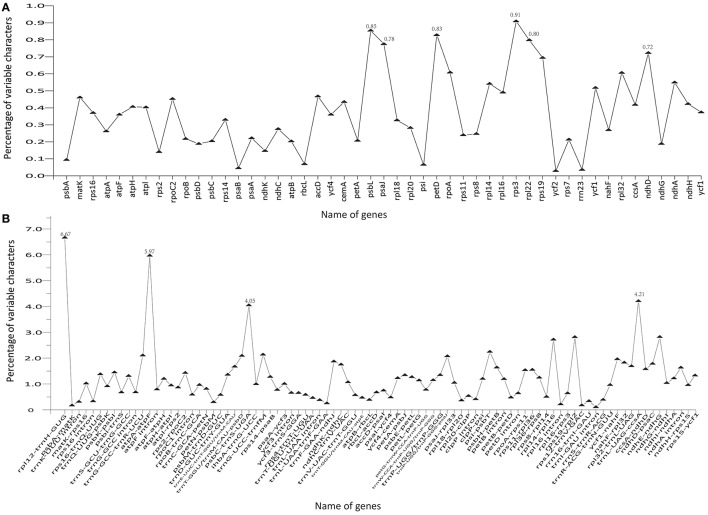
**Comparison of percentage of variable characters (SNPs, indels, and mutations) in five aligned ***Juglans*** chloroplast genomes. (A)** Protein coding sequences (CDS); **(B)** The introns and spacers (IGS).

The coding genes, non-coding regions, and introns were compared among the five Chinese *Juglans* species for divergence hotspots. The level of sequence divergence among all five species was estimated as the nucleotide variability value (*Pi* = 0.00219).The number of parsimony informative sites incoding genes, non-coding regions, and the complete Cpg was 192, 342, and 534, respectively (Table S7). The protein-coding CDS region was much more conserved than the IGS regions (i.e., LSC and SSC is much more conserved than the IR region). Within the CDS region, the ten genes with the greatest variability were *rps3, psbL, petD, rpl22, psaJ, ndhD, rps19, rpoA, rpl32*, and *ndhA* (Figure 5A), and the twelve least variable genes in CDS were *petA, psbC, atpB, psbD, ndhG, ndhK, rps2, psbA, rbcL, psi, psaB, rrn23*, and *ycf2* (Figure 5A). Some IGS were quite conserved; *rpl12-trnH-*GUG, *atpA-atpF, trnL-UAG-ccsA, psbC-trnS-*UGA, *ndhE*-*ndhG, rps19-rpl2, rpl14-rpl16, psi-psbT, ihbA-trnG-UC*C, *trnG-GCC-trnR-UCU, trnT-GGU/trnM-CAU-psbD*, and *trnP-UGG/trnP-GGG-psaJ* showed lower levels of variation than genes located in the CDS region (Figure 5B). Across all five species, the regions with greatest sequence divergence were *rps16*-*trnQ*-*UUG, trnE*-*UUC*-*trnT*-*GGU, trnT*-*GGU*-*psbD, petN*-*psbM, petB* intron, *rpoC2, ndhA*, and *ycf1*. These intergenic regions were also generally rich in SSRs; *rps16-trnQ-UUG*had four SSRs [(T)_10_, (A)_10_, (T)_11_, and (A)_11_]; *trnE-UUC-trnT-GGU* had three SSRs [(T)_10_, (A)_11_, and (AT)_7_]; *trnT*-*GGU*-*psbD* had one SSR [(AT)_6_]; *petN*-*psbM*, one SSR [(T)_10_]; *petB* intron, two SSRs[(A)_10_ and (A)_10_]; *rpoC2*, three SSRs [(T)_11_, (T)_11_, (T)_11_]; *ndhA* intron, four SSRs [(A)_15_, (T)13aattg…(T)_11_, (AT)_6_]; and *ycf1* had six SSRs [(T)_11_, (T)_10_, (T)_12_, (A)_10_, and (T)_12_. Within section *Juglans/Dioscaryon, rps4*-*trnT*-*UGU* (1 SNP), *ndhC*-*trnV*-*UAC* (1 SNP), *ycf1* (1 SNP; IRa), *ccsA*-*ndhD, ycf1* (3 SNP; IRb) were variable. Within section *Cardiocaryon, trnC*-*GCA*-*petN, trnE*-*UUC*-*trnT*-*GGU, trnT*-*GGU*-*psbD*, and *trnF*-*GAA*-*ndhJ* were most variable (Figure 3). In total, we identified 610 SNPs or indels that were distinct between *Juglans/Dioscaryon* and *Cardiocaryon*.

### Selective pressures in the evolution of *Juglans*

A total of 79 protein-coding genes were used to analyze synonymous and nonsynonymous change rates in *Juglans*. We identified five genes (*matK, ycf1, accD, rps3*, and *rpoA*) under positive selection (*K*_*A*_/*K*_*S*_ ratio >1; Figure S3; Table S8). The *K*_*A*_/*K*_*S*_ ratio for *accD* for all five species was 1.23. The *K*_*A*_/*K*_*S*_ ratio for *matK* for all five species was 1.34, for *rpoA* it was 1.17, and for *rps3* it was 1.38 (Table S8). Interestingly, these five genes were previously found to present above average SNV and indel densities in exons (Table S8). All five genes were under positive pressure exclusively between sect. *Cardiocaryon* and sect. *Dioscaryon*; none of these five genes showed evidence of positive selection within either section (Figure S3; Table S8).

### Phylogenetic analysis

**Figure 6:**
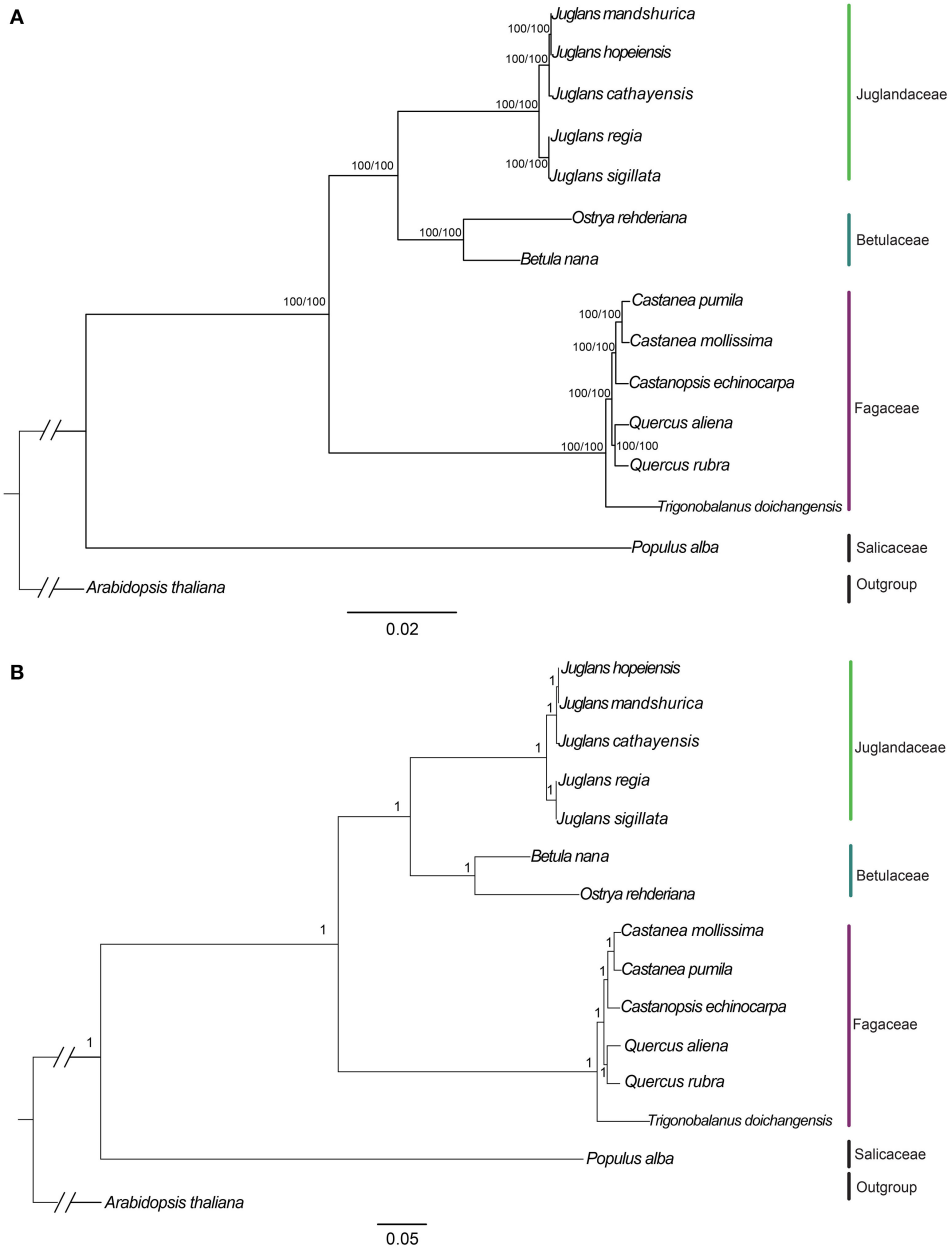
**Phylogeny of five ***Juglans*** species plus 8 taxa using (A)** Maximum Likelihood (ML) and **(B)** Bayesian inference (BI) based on whole cp genome sequences. Diagonal hash marks nested inside *Arabidopsis thaliana* represent a branch length truncation of 3/4. Numbers above branches are bootstrap support values.

We used three datasets (whole complete Cpg, protein-coding exons, and non-coding region) to analyze the phylogenetic relationships among members of two sections of *Juglans* and closely related species in the Betulaceae and Fagaceae. *Arabidopsis thaliana* and *Populus alba* were used as outgroups. Among the three datasets, complete Cpgs contained the greatest number of parsimony informative characters (531, 0.33%), followed by no-coding region (342, 0.42%) and protein-coding exons (192, 0.24%). The reconstructed phylogeny divided into four clades (Figure 6; Figures S4, S5, with members of the Betulaceae (*Ostrya rehderiana* and *Betula nana*) joined to the five *Juglans* species and distinct from the other Fagaceae, irrespective of dataset. Within *Juglans*, the five Chinese species were divided into two clades corresponding to the two sections (*Juglans/Dioscaryon* and *Cardiocaryon*) with 100 % bootstrap (BS)support based on Maximum Likelihood (ML) and Maximum parsimony (MP) analysis (Figure 6A; Figures S4A,B). Analysis of the whole cp genomes of the five Chinese walnut species and 10 eudicot outgroups using Bayesian inference (BI) resulted in cladograms with topology similar to ML and MP, and strongly supported phylognetic trees based on each of three datasets (whole cp genome sequences, protein coding sequences, and the introns and spacers) (Figure 6B; Figures S4C,D). In section *Juglans/Dioscaryon, J. regia* and *J. sigillata* were split with a 100% BS, while the *Cardiocaryon* clade (*J. cathayensis* and *J. hopeiensis, J. mandshurica*) diverged from sect. *Juglans* with 100% BS value (Figure 6; Figures S4, S5). *J. hopeiensis* was closer to *J. mandshurica* than to *J. cathayensis* (Figure 6; Figures S4, S5. We constructed the divergence time tree among five Chinese walnut species based on whole chloroplast genome sequences. The results showed that the divergence time between two sections was 7.91Myr, while *J. regia* and *J. sigillata* diverged much more recently (0.05 Myr), and *J. cathayensis* diverged from *J. mandshurica* and *J. hopeiensis* before 3.51Myr (Figure S5).

## Discussion

### Chloroplast sequence variation and evolution

In the present study, we sequenced the chloroplast genomes of five *Juglans* species, annotated the chloroplast genomes, identified SSR and tandem repeats within the genomes, and carried out a phylogenetic analysis comparing them to ten other chloroplast genomes. Our results have laid the foundation for future studies on the evolution of chloroplast genomes of walnuts and butternuts, as well as the molecular identification of *Juglans* species.

Most angiosperm chloroplasts contain 74 protein-coding genes, while an additional five are present in few species (Millen et al., 2001). The five *Juglans* Cpg we sequenced revealed 88 protein-coding genes (79 unigenes were protein-coding), 40 tRNA genes, and 8 rRNA genes, which is similar to *Quercus* (Du et al., 2015; Lu et al., 2016; Yang et al., 2016). The number of tRNA genes and rRNA genes in *Juglans* was the same as in five *Quercus* species (Yang et al., 2016). Moreover, the total number of introns in the *Juglans* Cpg was the same as *Quercus rubra* (Alexander and Woeste, 2014), *Ampelopsis* (Raman and Park, 2016), and Saxifragales (Dong et al., 2013). Several lineages of angiosperms have independently lost introns from the ribosomal protein genes *rps16, rps12*, and *rpl16* (Downie et al., 1991; Downie and Palmer, 1992), including Geraniaceae and Caryophyllales (Logacheva et al., 2008). The five Chinese *Juglans* species have not lost introns in any of these genes, however, a characteristic they have in common with the woody plant family Vitaceae (Raman and Park, 2016).

The gene *infA* encodes translation initiation factor 1. It has been lost completely in some angiosperms (Millen et al., 2001; Steane, 2005), is present as a pseudogenein the majority of angiosperm (Millen et al., 2001; Steane, 2005), and is present and presumed functional in *Quercus robur* and *Quercusrubra* (Alexander and Woeste, 2014). In this study, we identified nine internal stop codons in *Juglans/Dioscaryon* versus five, five, and three internal stop codons in the *infA* sequence of *J. hopeienis, J. mandshuria*, and *J. cathayensis* Cpgs, respectively. Thus, although *infA* is a pseudogene in all *Juglans/Dioscaryon* and *Cardiocaryon* for which there are data, there are inter-sectional differences that deserve additional study (Figure 2A), and *infA* may reveal important phylogenetic information concerning section *Rhysocaryon*. We also observed that the hypothetical gene *ycf15* was truncated in *Dioscaryon* species and *Cardiocaryon* species by five and three internal stop codons, respectively (Figure 2B). A similar truncation was seen in *Quercus aliena* (Lu et al., 2016, *ycf15*) and *Quercus spinosa* (Du et al., 2015) of Fagaceae, in Liliales (Liu et al., 2012b), Kiwi fruit (*Actinidia chinensis* var. chinensis) (Yao and Huang, 2016), and *Vaccinium macrocarpon* (Fajardo et al., 2013). *ycf15* is a pseudogene in all families of Saxifragales (Dong et al., 2013), but may be a functional protein coding gene in *Thalictrum coreanum* (Ranunculaceae, Park et al., 2015). The role of *ycf15* as a protein coding gene remains unclear and requires further study.

Variability in copy number of simple sequence repeats (SSRs) in the chloroplast makes them important molecular markers for distinguishing lower taxonomic levels (Yang et al., 2011; Xue et al., 2012). Cp SSRs have been used widely in plant population genetics (Doorduin et al., 2011; He et al., 2012), polymorphism investigations (Xue et al., 2012), and ecological and evolutionary studies (Roullier et al., 2011; Wang et al., 2013). The SSRs in the five *Juglans* Cp genomes we investigated were AT rich. Poly (A)/(T) SSRs are more common than poly (G)/(C) in many plant families (Melotto-Passarin et al., 2011; Nie et al., 2012; Martin et al., 2013). The cpSSRs of the five *Juglans* we studied are expected to be useful for assays detecting polymorphisms at population-level as well as comparing more distantly phylogenetic relationships among *Juglans* species.

Large and complex repeat sequences may play an important role chloroplast genome arrangement and sequence divergence (Timme et al., 2007; Guisinger et al., 2011; Weng et al., 2013). We found numerous repeated sequences in the Cpgs of *Juglans*, particularly in the intergenic spacer regions, similar to those reported in other angiosperm lineages (Yang et al., 2016). We found that repeats in *petB, psaA*, and *ycf2* differed between species in different sections of *Juglans*, and the same was true of repeats in the gene junctions (*trnK*-*UUU*-*rps16, trnV*-*GAC*-*rps7, trnT*-*GGU*-*psbD*, and *trnT*-*GGU*-*psbD*) (Table S5). These divergence hotspots within *Juglans* Cpg sequences are potentially important resources for developing molecular markers for phylogenetic analyses and identification of *Juglans* species (Stanford et al., 2000; Aradhya et al., 2007).

### Phylogenetic analysis

The classical taxonomy of *Juglans* based on non-coding regions of the Cpg supported the separation of *J. regia* and *J. sigillata* into Sec. *Juglans/Dioscaryon* and other three *Juglans* species (*J. cathayensis, J. hopeiensis, J. mandshurica*) into Sec. *Cardiocaryon* (Stanford et al., 2000; Aradhya et al., 2007). Whether *J. regia* and *J. sigillata* are legitimately distinct taxa in China has been controversial; Iron walnut (*J. sigillata*) could be an independent species based on RAPD and EST-SSR data (Wu et al., 2000; Qi et al., 2011) and based on RFLP and Cp DNA fragments(92% bootstrap value) (Aradhya et al., 2007). Our data support their maintenance as distinct taxa.

Members of the *Cardiocaryon* are morphologically distinct from other *Juglans* in that they have red stigmas, number of leaflets per leaf, and in the number of fruits typically found in a cluster, but the phylogenetic relationships within sect. *Cardiocaryon* are unsettled. *J. hopeiensis* is sympatric with *J. mandshurica*, and based on data from AFLPs and isozymes, some have concluded that *J. hopeiensis* is a hybrid species between *J. regia* and *J. mandshurica* (Wenheng, 1987; Zhang et al., 2009), consistent with the interpretation of floral evolution in the genus by Xi (1987). All phylogenetic trees based on our data indicate that *J. hopeiensis* is closer to *J. mandshurica* than *J. cathayensis*, and that the latter two species are distinct, in contrast to the Flora of China (1999), which relies exclusively on morphological data. The relationship between *J. hopeiensis* and *J. ailantifolia*, the only other Asian member of the *Cardiocaryon*, is now an important question. These results showed that the Stanford et al. (2000) and Aradhya et al. (2007) taxonomy of *Juglans* is reasonable on the whole. In this study, *J. regia* and *J. sigillata* were divided from each other with a 100% BS, while *J. cathayensis, J. hopeiensis*, and *J. mandshurica* diverged from sect. *Juglans* with 100% BS value (Figure 6. Each of the five species is supported as independent species based on whole chloroplast genome sequences.

In this study, the five Chinese walnut species and 10 eudicot outgroups were represented with well-supported cladograms with highly similar topology and strongly supported phylogenetic trees using Maximum Likelihood (ML), Bayesian inference (BI), and Maximum parsimony (MP) analysis. Analysis using whole Cpg sequences, protein coding sequences, and the introns and spacers resulted in consistent and strongly supported results (Figure 6; Figure S4). Our results confirmed that the phylogenetic relationships among the five Chinese *Juglans* based on chloroplast sequences only are in congruence with those reported by Stanford et al. (2000) and Aradhya et al. (2007). Each of the two sections was confirmed to be monophyletic (Dode, 1909; Manning, 1978). Within sect. *Dioscaryon*, division of the two species was highly supported, as suggested by Aradhya et al. (2007). With the exception of section *Cardiocaryon* (Dode, 1909; Manning, 1978), relationships among three Chinese walnuts were fully resolved and statistically supported (*P* = 0.95; BS = 100%). Stanford et al. (2000) and Aradhya et al. (2007) recovered an unsupported sister relationship between *J. mandshurica* and *J. cathayensis* because *J. hopeiensis* was not included in those analyses (Stanford et al., 2000). Previously suggested relationships among members of section *Cardiocaryon* were confirmed by our data with even higher support than in Stanford et al. (2000) and Aradhya et al. (2007), although our analysis did not include Japanese walnut (*J. ailantifolia*), the final member of *Cardiocaryon*. The chloroplast-based phylogeny presented in this work and by others is not a complete understanding of the evolutionary relationships among these five Chinese *Juglans* because events we did not consider, including incomplete lineage sorting, chloroplast capture, horizontal transfer, and local fixation of cpG haplotypes can all influence phylogeny (Stegemann et al., 2012; Mariac et al., 2014; Novikova et al., 2016).

The divergence time between the two Asian *Juglans* sections was estimated at 7.91Myr, although several *Juglans* species diverged quite recently within each section (Figure 6; Figure S4). The deep evolutionary relationships and divisions within the two Asian sections needs further investigation. The molecular phylogeny of the entire genus (*Juglans*) and its relationship to other genera in the Juglandaceae also awaits more evidence. These Cpg sequences will provide genetic information necessary to understand the evolution of plastid genomes via phylogenomics.

## Data archiving statement

The chloroplast genome sequences of Chinese walnut (*Juglans*) species were submitted on the National Center for Biotechnology Information (NCBI), the accession numbers were: KT820730, KT820731, and KT820732, KT820733.

## Ethics statement

This article does not contain any studies with human participants performed by any of the authors.

## Author contributions

PZ, YH, and KW designed and performed the experiment as well as drafted the manuscript. YH and PZ collected the samples. YH and PZ completed the sequence assembly and analyzed the data. KW and PZ conceived the study and revised the manuscript. All the authors have read and approved the final manuscript.

### Conflict of interest statement

The authors declare that the research was conducted in the absence of any commercial or financial relationships that could be construed as a potential conflict of interest.

## Supplementary material

Figure S1 **Comparisons of LSC, SSC, and IR region borders among the five Chinese ***Juglans*** chloroplast genomes**.

Figure S2 **Frequency distribution of major SSRs based on main motif type in the five Chinese ***Juglans*** cp genomes**. Jh, *Juglans hopeiensis*; Jc, *J. cathayensis*; Jm, *J. mandshurica*; Jr, *J. regia*; Js, *J. sigillata*.

Figure S3 **Gene-specific ***K***_***A***_/***K***_***S***_ values between the chloroplast genomes of two ***Juglans***species (***J. regia*** and ***J. cathayensis***) representing section ***Juglans/Dioscaryon*** and section ***Cardiocaryon***, respectively**. Five genes (*matK, ycf1, accD, rps3*, and *rpoA*) returned *K*_*A*_/*K*_*S*_ values greater than 0.8, whereas the *K*_*A*_/*K*_*S*_ values of the other genes were below 0.8.

Figure S4 **Phylogenetic tree construction of five ***Juglans*** species plus eight other taxa. (A)** Maximum Likelihood (ML) tree and Maximum parsimony (MP) tree based on protein coding sequences, **(B)** Maximum Likelihood (ML) tree and Maximum parsimony (MP)tree based on the introns and spacers, **(C)** Bayesian inference (BI)tree based on protein coding sequences, **(D)** Bayesian inference (BI) treebased on the introns and spacers. Numbers above branch indicate the bootstrap (BS) support value.

Figure S5 **Phylogenetic timetree construction of five Chinese ***Juglans*** species plus eight other taxa based on whole cp genome sequences**. Blue bars and the numbers at the nodes indicate 95% highest posterior densities (HPDs) of time estimates (million years ago, Myr).

Table S1 **Primers used for genome sequence validation**.

Table S2 **The information of a total of 15 species used for phylogenetic analysis**.

Table S3 **Indels and single nucleotide polymorphisms (SNP) in the five Chinese ***Juglans*** chloroplast genomes**.

Table S4 **Simple sequence repeats in each of five Chinese ***Juglans*** species**.

Table S5 **The information of the function nucleic acid repeats of five Chinese ***Juglans*** species**.

Table S6 **The length of tandem repeats distribution in five Chinese ***Juglans*** species**.

Table S7 **The number of variable sites in five Chinese ***Juglans*** species**.

Table S8 *****K***_***A***_/***K***_***S***_ ratio for protein coding sequences for five Chinese ***Juglans*** species**. Jh, *Juglans hopeiensis*; Jc, *J. cathayensis*; Jm, *J. mandshurica*; Jr, *J. regia*; Js, *J. sigillata*.
