# Supplementary figures and images for: Completion of the Chloroplast Genomes of Five Chinese Juglans and Their Contribution to Chloroplast Phylogeny
Source: Front Plant Sci. 2017 Jan 6;7:1955. doi: 10.3389/fpls.2016.01955 (PMC5216037; doi:10.3389/fpls.2016.01955)

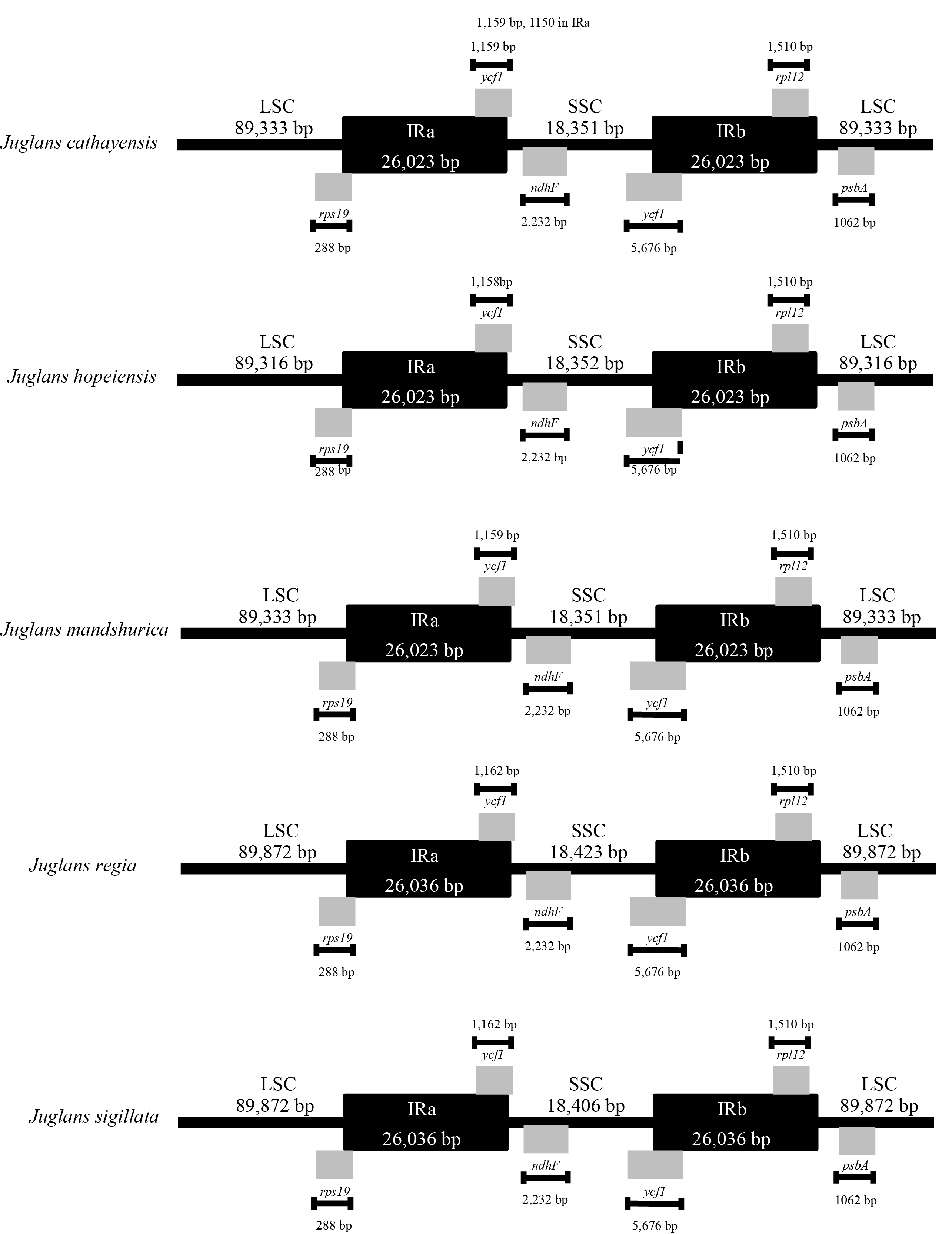

Supplement: Figure S1 — Comparisons of LSC, SSC, and IR region borders among the five Chinese Juglans chloroplast genomes. [file Image1.JPEG]

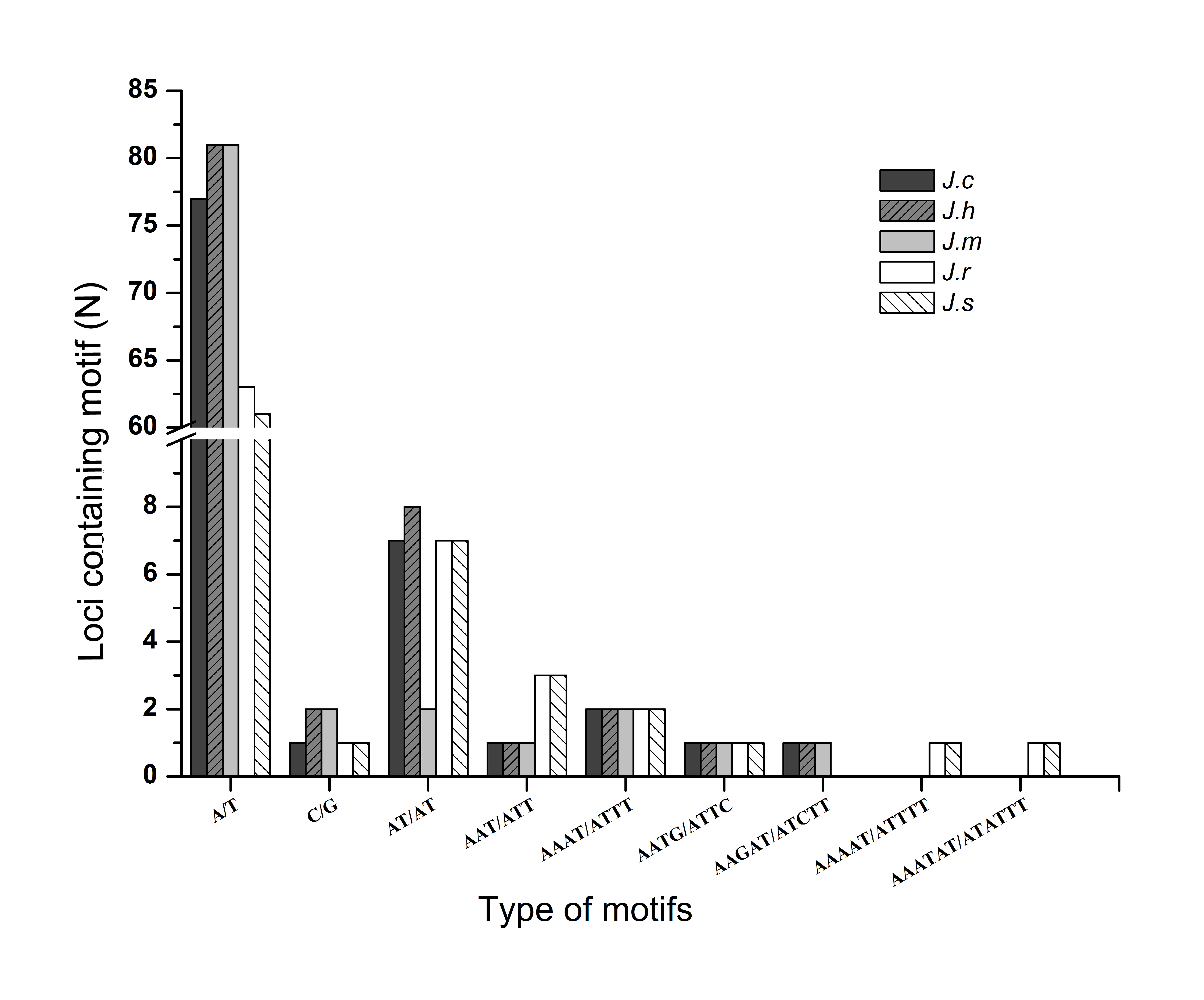

Supplement: Figure S2 — Frequency distribution of major SSRs based on main motif type in the five Chinese Juglans cp genomes. Jh, Juglans hopeiensis; Jc, J. cathayensis; Jm, J. mandshurica; Jr, J. regia; Js, J. sigillata. [file Image2.JPEG]

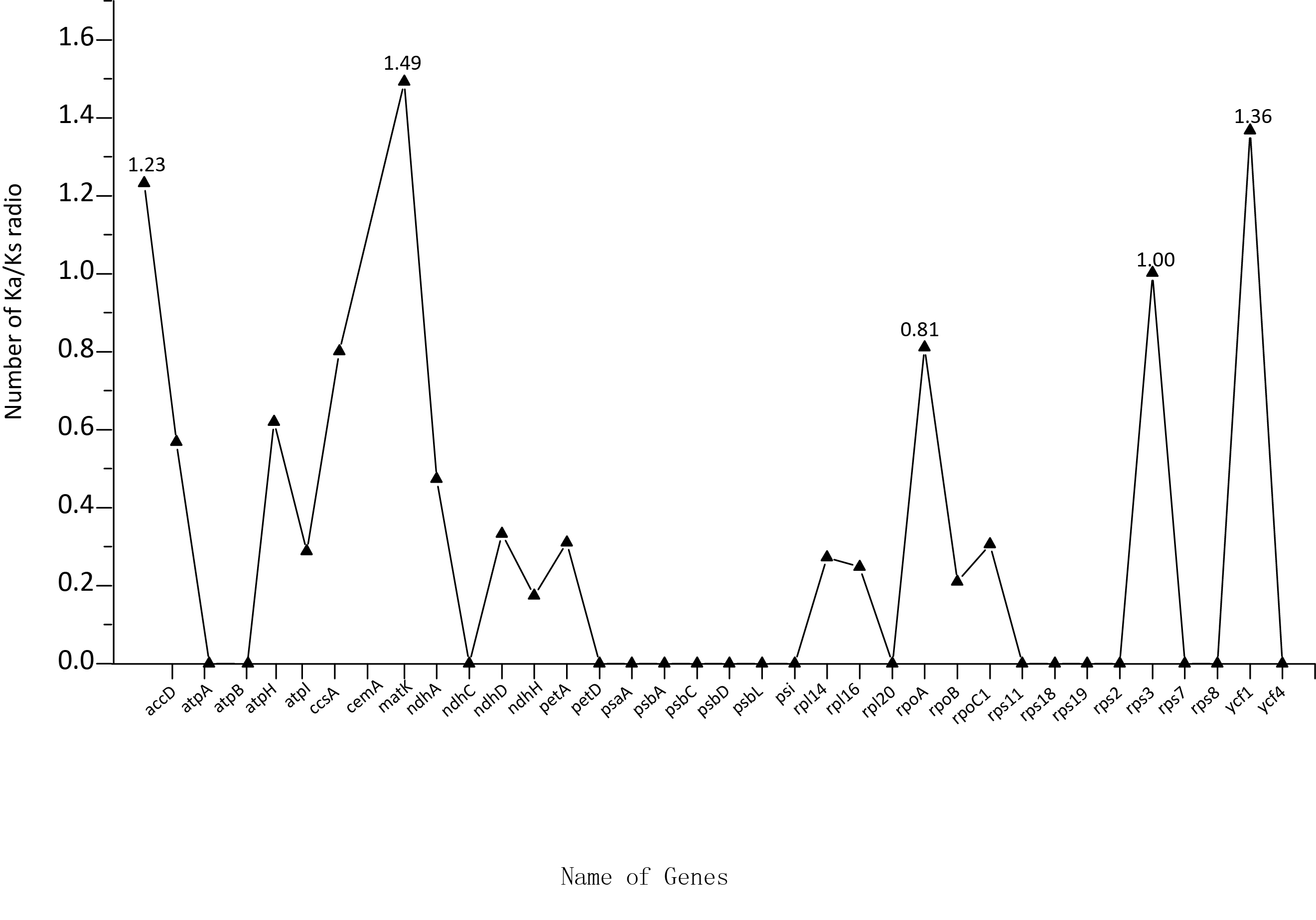

Supplement: Figure S3 — Gene-specific KA/KS values between the chloroplast genomes of two Juglansspecies (J. regia and J. cathayensis) representing section Juglans/Dioscaryon and section Cardiocaryon, respectively. Five genes (matK, ycf1, accD, rps3, and rpoA) returned KA/KS values greater than 0.8, whereas the KA/KS values of the other genes were below 0.8. [file Image3.JPEG]

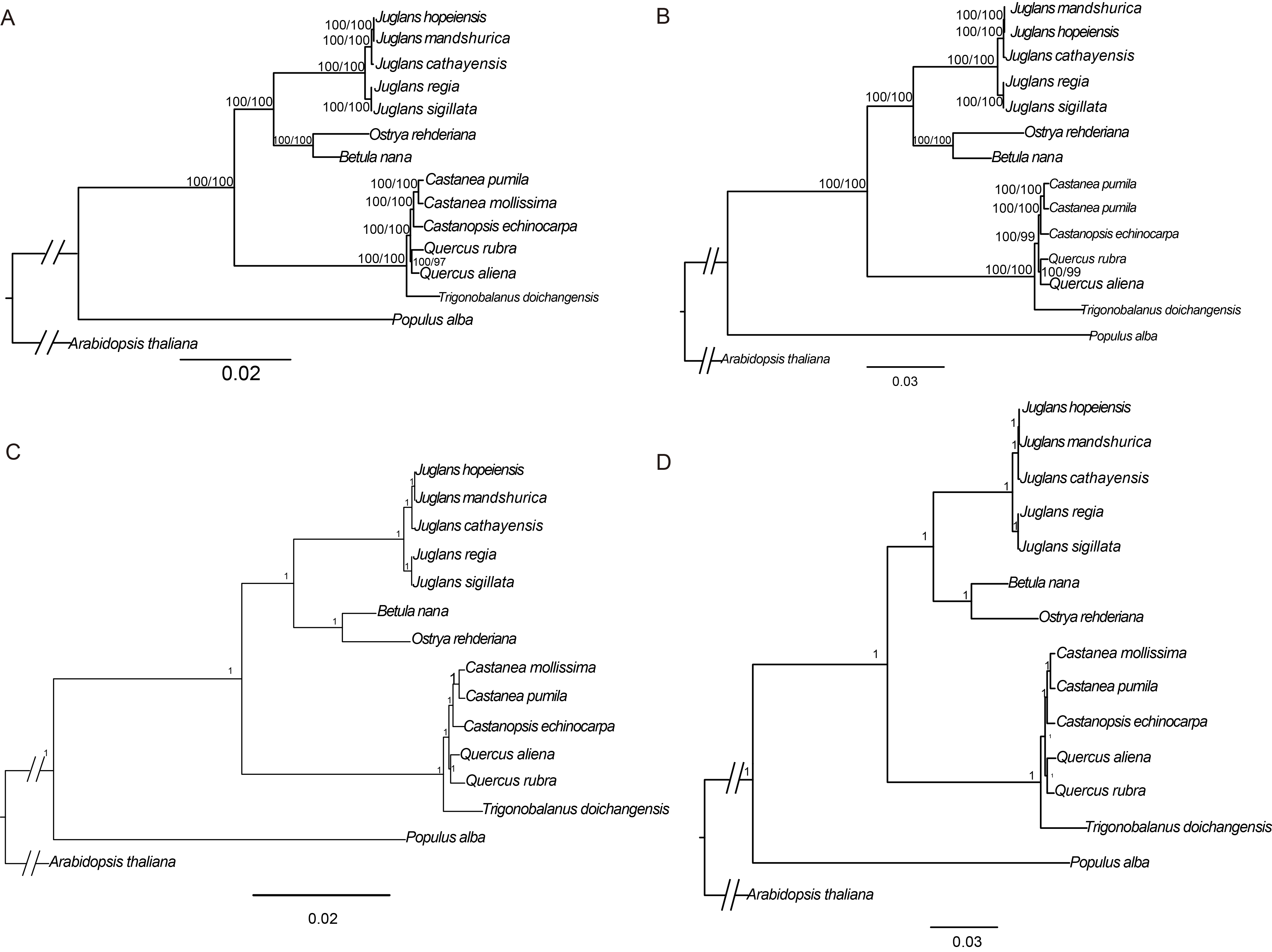

Supplement: Figure S4 — Phylogenetic tree construction of five Juglans species plus eight other taxa. (A) Maximum Likelihood (ML) tree and Maximum parsimony (MP) tree based on protein coding sequences, (B) Maximum Likelihood (ML) tree and Maximum parsimony (MP)tree based on the introns and spacers, (C) Bayesian inference (BI)tree based on protein coding sequences, (D) Bayesian inference (BI) treebased on the introns and spacers. Numbers above branch indicate the bootstrap (BS) support value. [file Image4.JPEG]

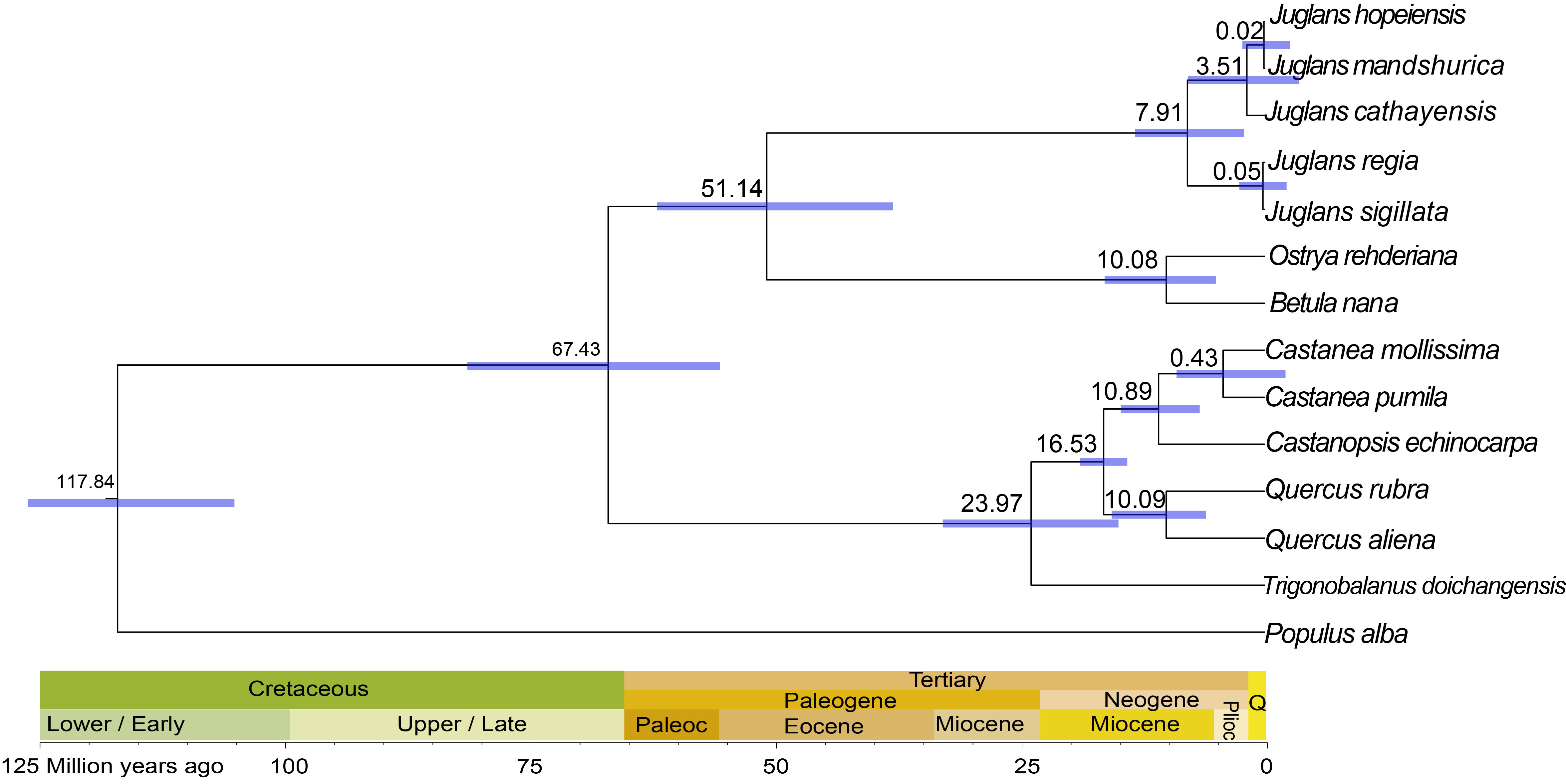

Supplement: Figure S5 — Phylogenetic timetree construction of five Chinese Juglans species plus eight other taxa based on whole cp genome sequences. Blue bars and the numbers at the nodes indicate 95% highest posterior densities (HPDs) of time estimates (million years ago, Myr). [file Image5.JPEG]
