# Supplementary material for: Completion of the Chloroplast Genomes of Five Chinese Juglans and Their Contribution to Chloroplast Phylogeny
Source: Front Plant Sci. 2017 Jan 6;7:1955. doi: 10.3389/fpls.2016.01955 (PMC5216037; doi:10.3389/fpls.2016.01955)
Supplement: Table S1 — Primers used for genome sequence validation. [file Table1.DOC]

**Table S1. Primers used for genome sequence validation**

| Locus Name | Primer sequence 5'to3' | Tm |
| --- | --- | --- |
| cp-link-F | CGGACAAGTGGGGAATGTTG | 60 |
| cp-link-R | AAGTGGATCAAGGCAGTGGA |  |
| J31775-F | TCAACGGTGTTGGGTTAGAA | 60 |
| J31775-R | TGAATCCCGATATCCATTGAA |  |
| J36659-F | GGCTCTGTAAGAATTCGTCGAT | 60 |
| J36659-R | CCCCAGACATATTCCTCTCG |  |
| J41387-F | TGGCCTAGCTCTGTCCAAAT | 60 |
| J41387-R | CACGTCTTCATCGACGTTGT |  |
| J50821-F | TTTTGTTTACCGAGGGTTCG | 60 |
| J50821-R | GGAAGCGACCTTAGAAACCA |  |
| J58012-F | TTTGGGATGATAAATGACCTACTT | 60 |
| J58012-R | TGAAGAAATGACCTTAAATCTTTGTG |  |
| J70476-F | CGTTGTATGAACTGCATTGCT | 60 |
| J70476-R | CCGAAGCCAATAATGGAGAG |  |
| J81653-F | CGGTTGGGGAACTACTCCTT | 60 |
| J81653-R | TTGATGGTTACAGGCCTCTTG |  |
| J116758-F | TCCGAAACAAAGGGGACTAA | 60 |
| J116758-R | CAATCGAATTCTTTGGGAAAA |  |
| J122347-F | CGAAAGAAACTTGGGCATTT | 60 |
| J122347-R | GGGGATGAGTGGTTTTGTTG |  |
| J127629-F | CACCTCATACGGCTCCCTTA | 60 |
| J127629-R | GACGGTTAGAAACGCCAAAG |  |

Note: The first two letters in each primer’s ID represent the names of the link LSC and IRb, and the gap between the sequences was filled by this primer. F and R represent “Forward” and “Reverse,” respectively.
