# Supplementary material for: Completion of the Chloroplast Genomes of Five Chinese Juglans and Their Contribution to Chloroplast Phylogeny
Source: Front Plant Sci. 2017 Jan 6;7:1955. doi: 10.3389/fpls.2016.01955 (PMC5216037; doi:10.3389/fpls.2016.01955)
Supplement: Table S2 — The information of a total of 15 species used for phylogenetic analysis. [file Table2.DOC]

**Table S2. The information of a total of 15 species used for phylogenetic analysis**

| Order | Taxon | Genbank Accession number | Family |
| --- | --- | --- | --- |
| 1 | *Juglans cathayensis* | KX671976 | Juglandaceae |
| 2 | *Juglans hopeiensis* | KX671977 | Juglandaceae |
| 3 | *Juglans mandshurica* | KX671975 | Juglandaceae |
| 4 | *Juglans regia* | KT963008 | Juglandaceae |
| 5 | *Juglans sigillata* | KX424843 | Juglandaceae |
| 6 | *Ostrya rehderiana* | NC_028349 | Betulaceae |
| 7 | *Betula nana* | KX703002 | Betulaceae |
| 8 | *Castanea mollissima* | NC_014674 | Fagaceae |
| 9 | *Castanea pumila* | KM360048 | Fagaceae |
| 10 | *Castanopsis echinocarpa* | NC_023801 | Fagaceae |
| 11 | *Quercus aliena* | NC_026790 | Fagaceae |
| 12 | *Quercus rubra* | NC_020152 | Fagaceae |
| 13 | *Trigonobalanus doichangensis* | NC_023959 | Fagaceae |
| 14 | *Populus alba* | NC_008235 | [Salicaceae](https://en.wikipedia.org/wiki/Salicaceae) |
| 15 | *Arabidopsis thaliana* | NC_000932 | [Brassicaceae](https://en.wikipedia.org/wiki/Brassicaceae) |
