# Supplementary material for: Completion of the Chloroplast Genomes of Five Chinese Juglans and Their Contribution to Chloroplast Phylogeny
Source: Front Plant Sci. 2017 Jan 6;7:1955. doi: 10.3389/fpls.2016.01955 (PMC5216037; doi:10.3389/fpls.2016.01955)
Supplement: Table S3 — Indels and single nucleotide polymorphisms (SNP) in the five Chinese Juglans chloroplast genomes. [file Table3.DOC]

**Table S3**. Indel and single nucleotide polymorphisms (SNP) in the five Chinese *Juglans* chloroplast genomes.

| Type | position | *J. cathayensis* | *J. hopeiensis* | *J. mandshurica* | *J. regia* | *J. sigillata* |
| --- | --- | --- | --- | --- | --- | --- |
| SNP | 7 | A | - | - | T | T |
| SNP | 8 | A | - | - | T | T |
| SNP | 1089 | A | G | G | G | G |
| SNP | 1965 | C | C | C | T | T |
| SNP | 2207 | T | G | G | T | T |
| SNP | 2209 | A | G | G | A | A |
| SNP | 2673 | A | A | A | G | G |
| SNP | 2718 | A | A | A | T | T |
| SNP | 3226 | G | G | G | A | A |
| SNP | 3350 | C | C | C | T | T |
| SNP | 3473 | T | T | T | A | A |
| SNP | 3505 | G | G | G | T | T |
| SNP | 3745 | G | G | G | T | T |
| SNP | 4015 | T | T | T | C | C |
| SNP | 4061 | C | C | C | T | T |
| SNP | 4307 | C | C | C | A | A |
| SNP | 4333 | T | G | G | G | G |
| SNP | 4383 | C | C | C | A | A |
| SNP | 4700 | C | C | C | A | A |
| SNP | 4752 | T | T | T | G | G |
| SNP | 4763 | A | A | A | T | T |
| SNP | 4764 | A | A | A | T | T |
| SNP | 4765 | A | A | A | T | T |
| SNP | 5008 | T | T | T | G | G |
| SNP | 5059 | A | A | A | G | G |
| SNP | 5128 | A | A | A | T | T |
| SNP | 5181 | G | G | G | T | T |
| SNP | 5303 | C | C | C | T | T |
| SNP | 5307 | T | C | C | C | C |
| SNP | 5308 | T | C | C | C | C |
| SNP | 5464 | C | C | C | A | A |
| SNP | 5612 | C | C | C | T | T |
| SNP | 5660 | T | T | T | G | G |
| SNP | 6312 | A | C | C | C | C |
| SNP | 6566 | T | T | T | C | C |
| SNP | 6688 | C | C | C | A | A |
| SNP | 6758 | C | C | C | T | T |
| SNP | 7143 | G | G | G | A | A |
| SNP | 7417 | C | C | C | T | T |
| SNP | 7503 | T | T | T | A | A |
| SNP | 7511 | A | A | A | T | T |
| SNP | 7532 | G | G | G | A | A |
| SNP | 7775 | T | T | T | G | G |
| SNP | 7838 | A | A | A | G | G |
| SNP | 7862 | T | T | T | A | A |
| SNP | 7906 | T | T | T | A | A |
| SNP | 7907 | T | A | A | A | A |
| SNP | 8162 | A | A | A | G | G |
| SNP | 8361 | A | A | A | T | T |
| SNP | 8713 | A | A | A | T | T |
| SNP | 8776 | A | A | A | C | C |
| SNP | 8791 | C | C | C | G | G |
| SNP | 8973 | T | T | T | G | G |
| SNP | 9020 | C | A | A | C | C |
| SNP | 9503 | G | A | A | G | G |
| SNP | 9590 | A | A | A | G | G |
| SNP | 9821 | T | T | T | A | A |
| SNP | 9847 | C | C | C | A | A |
| SNP | 9921 | A | A | A | C | C |
| SNP | 10049 | T | T | T | C | C |
| SNP | 10051 | T | T | T | G | G |
| SNP | 10114 | T | C | C | C | C |
| SNP | 10132 | C | C | C | A | A |
| SNP | 10358 | G | G | G | A | A |
| SNP | 10768 | A | A | A | C | C |
| SNP | 10901 | G | G | G | T | T |
| SNP | 11148 | A | A | A | T | T |
| SNP | 11243 | G | G | G | A | A |
| SNP | 11276 | A | A | A | T | T |
| SNP | 11302 | A | A | A | C | C |
| SNP | 11355 | G | G | G | A | A |
| SNP | 11944 | T | T | T | C | C |
| SNP | 12632 | C | T | T | C | C |
| SNP | 12749 | A | A | A | G | G |
| SNP | 13293 | G | G | G | A | A |
| SNP | 13328 | T | T | T | A | A |
| SNP | 13334 | T | G | G | T | T |
| SNP | 13335 | C | A | A | T | T |
| SNP | 13336 | A | A | A | T | T |
| SNP | 13337 | A | A | A | T | T |
| SNP | 13338 | A | A | A | T | T |
| SNP | 13339 | A | A | A | C | C |
| SNP | 13346 | T | T | T | A | A |
| SNP | 13644 | A | A | A | C | C |
| SNP | 13807 | C | C | C | T | T |
| SNP | 13909 | T | T | T | C | C |
| SNP | 14174 | G | T | T | T | T |
| SNP | 14639 | G | A | A | A | A |
| SNP | 14692 | C | C | C | A | A |
| SNP | 14810 | C | T | T | T | T |
| SNP | 15065 | G | G | G | T | T |
| SNP | 15099 | C | C | C | G | G |
| SNP | 15385 | T | T | T | C | C |
| SNP | 15697 | A | A | A | G | G |
| SNP | 15837 | T | T | T | G | G |
| SNP | 16051 | A | A | A | C | C |
| SNP | 16223 | T | T | T | C | C |
| SNP | 16304 | C | C | C | T | T |
| SNP | 16342 | A | A | A | G | G |
| SNP | 16473 | A | A | A | G | G |
| SNP | 16598 | G | G | G | T | T |
| SNP | 16872 | C | T | T | T | T |
| SNP | 16954 | T | T | T | C | C |
| SNP | 17381 | A | C | C | C | C |
| SNP | 17614 | C | C | C | T | T |
| SNP | 18120 | T | T | T | C | C |
| SNP | 18410 | T | A | A | A | A |
| SNP | 18411 | T | A | A | A | A |
| SNP | 18412 | T | A | A | A | A |
| SNP | 18594 | A | A | A | T | T |
| SNP | 18659 | T | T | T | G | G |
| SNP | 19473 | T | T | T | G | G |
| SNP | 19595 | C | C | C | T | T |
| SNP | 19631 | C | C | C | T | T |
| SNP | 19730 | A | A | A | G | G |
| SNP | 19943 | C | C | C | T | T |
| SNP | 20287 | A | C | C | A | A |
| SNP | 20331 | A | A | A | G | G |
| SNP | 20490 | G | G | G | A | A |
| SNP | 20565 | A | A | A | G | G |
| SNP | 20587 | C | T | T | T | T |
| SNP | 21011 | A | G | G | G | G |
| SNP | 22588 | T | T | T | C | C |
| SNP | 23125 | G | G | G | A | A |
| SNP | 23215 | C | C | C | A | A |
| SNP | 23632 | T | T | T | G | G |
| SNP | 23688 | T | T | T | C | C |
| SNP | 24722 | C | C | C | A | A |
| SNP | 24878 | A | A | A | T | T |
| SNP | 26323 | C | C | C | T | T |
| SNP | 27353 | A | A | A | G | G |
| SNP | 27615 | G | G | G | A | A |
| SNP | 27745 | T | G | G | G | G |
| SNP | 27772 | C | C | C | T | T |
| SNP | 28548 | G | G | G | T | T |
| SNP | 29077 | G | G | G | A | A |
| SNP | 29186 | T | T | T | G | G |
| SNP | 29579 | A | A | A | G | G |
| SNP | 29723 | C | C | C | T | T |
| SNP | 29764 | G | G | G | T | T |
| SNP | 29789 | A | A | A | T | T |
| SNP | 29822 | A | A | A | C | C |
| SNP | 30130 | T | T | T | G | G |
| SNP | 30183 | G | G | G | A | A |
| SNP | 30197 | A | A | A | G | G |
| SNP | 30215 | G | G | G | A | A |
| SNP | 30311 | A | C | C | - | - |
| SNP | 30485 | T | T | T | A | A |
| SNP | 30678 | A | A | A | G | G |
| SNP | 30690 | A | G | G | G | G |
| SNP | 30810 | T | T | T | C | C |
| SNP | 30974 | A | A | A | G | G |
| SNP | 31018 | G | G | G | A | A |
| SNP | 31977 | G | G | G | T | T |
| SNP | 32056 | T | T | T | A | A |
| SNP | 32546 | C | A | A | A | A |
| SNP | 33318 | A | A | A | G | G |
| SNP | 33351 | C | C | C | A | A |
| SNP | 33383 | A | A | A | G | G |
| SNP | 33632 | A | A | A | G | G |
| SNP | 33845 | T | A | A | T | T |
| SNP | 34031 | G | G | G | C | C |
| SNP | 34032 | A | A | A | T | T |
| SNP | 34033 | G | G | G | C | C |
| SNP | 34277 | A | A | A | G | G |
| SNP | 34630 | C | C | C | T | T |
| SNP | 34730 | A | A | A | T | T |
| SNP | 34732 | A | A | A | T | T |
| SNP | 34733 | G | G | G | A | A |
| SNP | 34736 | T | T | T | A | A |
| SNP | 34737 | C | C | C | T | T |
| SNP | 35087 | A | A | A | C | C |
| SNP | 35299 | C | C | C | T | T |
| SNP | 35345 | G | G | G | C | C |
| SNP | 35363 | T | T | T | C | C |
| SNP | 35402 | G | G | G | T | T |
| SNP | 35448 | G | G | G | A | A |
| SNP | 35656 | T | T | T | G | G |
| SNP | 35868 | T | T | T | G | G |
| SNP | 35972 | C | C | C | G | G |
| SNP | 36097 | A | A | A | G | G |
| SNP | 36135 | G | G | G | T | T |
| SNP | 36151 | T | T | T | C | C |
| SNP | 36169 | A | A | A | G | G |
| SNP | 36260 | G | G | G | T | T |
| SNP | 36286 | A | A | A | C | C |
| SNP | 36379 | A | A | A | T | T |
| SNP | 36383 | G | G | G | A | A |
| SNP | 36387 | - | T | - | G | G |
| SNP | 36415 | A | T | T | A | A |
| SNP | 36417 | T | T | T | A | A |
| SNP | 36516 | A | A | A | G | G |
| SNP | 36520 | C | C | C | T | T |
| SNP | 36541 | T | C | C | C | C |
| SNP | 36584 | A | A | A | T | T |
| SNP | 36586 | T | T | T | A | A |
| SNP | 36587 | T | T | T | A | A |
| SNP | 36589 | A | A | A | T | T |
| SNP | 36620 | A | A | A | T | T |
| SNP | 36702 | T | T | T | G | G |
| SNP | 36855 | T | T | T | G | G |
| SNP | 37095 | G | G | G | A | A |
| SNP | 37104 | A | A | A | G | G |
| SNP | 37768 | G | G | G | A | A |
| SNP | 37822 | A | A | A | C | C |
| SNP | 38393 | G | G | G | C | C |
| SNP | 39248 | C | C | C | T | T |
| SNP | 39416 | G | G | G | T | T |
| SNP | 39823 | C | C | C | T | T |
| SNP | 39863 | T | T | T | G | G |
| SNP | 39864 | C | C | C | G | G |
| SNP | 39865 | C | C | C | G | G |
| SNP | 39866 | C | C | C | G | G |
| SNP | 39867 | C | C | C | G | G |
| SNP | 39868 | C | C | C | G | G |
| SNP | 39869 | C | C | C | A | A |
| SNP | 39943 | A | A | A | C | C |
| SNP | 40096 | C | C | C | T | T |
| SNP | 40243 | T | T | T | C | C |
| SNP | 40291 | A | G | G | G | G |
| SNP | 40432 | A | G | G | G | G |
| SNP | 40741 | C | C | C | G | G |
| SNP | 40833 | C | C | C | A | A |
| SNP | 40835 | T | T | T | A | A |
| SNP | 40880 | T | T | T | A | A |
| SNP | 40905 | T | T | T | G | G |
| SNP | 40928 | A | A | A | G | G |
| SNP | 41755 | T | T | T | G | G |
| SNP | 41861 | C | A | A | C | C |
| SNP | 43769 | C | C | C | A | A |
| SNP | 44968 | G | G | G | A | A |
| SNP | 45145 | G | G | G | A | A |
| SNP | 45859 | C | T | T | C | C |
| SNP | 45898 | G | A | A | G | G |
| SNP | 46432 | T | T | T | G | G |
| SNP | 46703 | G | G | G | T | T |
| SNP | 46704 | A | A | A | C | C |
| SNP | 46828 | T | T | T | A | A |
| SNP | 46857 | A | G | G | G | G |
| SNP | 46859 | T | T | T | C | C |
| SNP | 46876 | C | C | C | T | T |
| SNP | 46923 | A | A | A | T | T |
| SNP | 47522 | T | G | G | G | G |
| SNP | 47683 | A | A | A | C | C |
| SNP | 47714 | C | C | C | T | T |
| SNP | 48513 | G | T | T | T | T |
| SNP | 48754 | T | C | C | C | C |
| SNP | 48834 | C | C | C | A | A |
| SNP | 48924 | A | A | A | G | G |
| SNP | 49077 | C | C | C | T | T |
| SNP | 49733 | G | G | G | A | A |
| SNP | 49943 | T | T | T | G | G |
| SNP | 50073 | T | T | T | C | C |
| SNP | 51166 | G | G | G | G | T |
| SNP | 51674 | G | C | C | C | C |
| SNP | 52318 | A | A | A | G | G |
| SNP | 52324 | A | A | A | G | G |
| SNP | 52496 | A | A | A | T | T |
| SNP | 52692 | T | T | T | C | C |
| SNP | 53011 | T | T | T | G | G |
| SNP | 53533 | T | T | T | C | C |
| SNP | 53576 | C | C | C | T | T |
| SNP | 53578 | A | T | T | T | T |
| SNP | 53820 | A | A | A | C | C |
| SNP | 54101 | T | T | T | G | G |
| SNP | 54182 | T | T | T | A | A |
| SNP | 54184 | C | C | C | A | A |
| SNP | 54187 | T | T | T | A | A |
| SNP | 54190 | T | T | T | A | A |
| SNP | 54211 | T | T | T | A | A |
| SNP | 55118 | T | T | T | C | C |
| SNP | 55858 | G | G | G | T | T |
| SNP | 56134 | G | G | G | T | T |
| SNP | 56326 | C | C | C | A | A |
| SNP | 56339 | A | A | A | C | C |
| SNP | 56397 | G | G | G | A | A |
| SNP | 56448 | T | T | T | T | A |
| SNP | 57411 | C | A | A | C | C |
| SNP | 58883 | T | T | T | G | G |
| SNP | 59219 | T | T | T | C | C |
| SNP | 59306 | A | A | A | G | G |
| SNP | 60193 | T | T | T | G | G |
| SNP | 60371 | T | T | T | G | G |
| SNP | 60394 | T | T | T | C | C |
| SNP | 61421 | T | T | T | G | G |
| SNP | 62028 | C | C | C | A | A |
| SNP | 62166 | A | A | A | C | C |
| SNP | 62346 | T | T | T | C | C |
| SNP | 62542 | G | G | G | T | T |
| SNP | 62553 | G | G | G | A | A |
| SNP | 62617 | T | T | T | C | C |
| SNP | 63011 | C | C | C | T | T |
| SNP | 63281 | C | C | C | A | A |
| SNP | 63345 | T | T | T | G | G |
| SNP | 63636 | C | C | C | A | A |
| SNP | 64094 | T | T | T | C | C |
| SNP | 64217 | A | T | T | A | A |
| SNP | 64634 | T | T | T | A | A |
| SNP | 65122 | C | C | C | T | T |
| SNP | 65613 | A | A | A | G | G |
| SNP | 65682 | C | C | C | T | T |
| SNP | 66030 | A | A | A | G | G |
| SNP | 66075 | G | A | A | G | G |
| SNP | 66121 | A | G | G | G | G |
| SNP | 66528 | T | T | T | C | C |
| SNP | 66580 | A | A | A | G | G |
| SNP | 67005 | T | T | T | A | A |
| SNP | 67566 | T | T | T | G | G |
| SNP | 67710 | G | G | G | A | A |
| SNP | 68371 | T | T | T | A | A |
| SNP | 68372 | A | A | A | T | T |
| SNP | 68424 | G | G | G | T | T |
| SNP | 68443 | T | T | T | C | C |
| SNP | 68933 | C | C | C | T | T |
| SNP | 68934 | C | C | C | T | T |
| SNP | 68935 | A | A | A | C | C |
| SNP | 68941 | A | A | A | G | G |
| SNP | 68942 | C | C | C | G | G |
| SNP | 68943 | C | C | C | T | T |
| SNP | 69743 | A | A | A | G | G |
| SNP | 70247 | T | T | T | G | G |
| SNP | 70711 | G | G | G | C | C |
| SNP | 70827 | A | A | A | G | G |
| SNP | 70847 | A | A | A | T | T |
| SNP | 70902 | A | G | G | G | G |
| SNP | 70912 | C | C | C | T | T |
| SNP | 71066 | G | G | G | T | T |
| SNP | 71134 | C | C | C | A | A |
| SNP | 71345 | C | C | C | A | A |
| SNP | 71360 | G | T | T | G | G |
| SNP | 71421 | T | T | T | C | C |
| SNP | 71436 | A | A | A | G | G |
| SNP | 71714 | C | C | C | A | A |
| SNP | 72109 | T | T | T | A | A |
| SNP | 72250 | G | G | G | T | T |
| SNP | 72340 | C | C | C | T | T |
| SNP | 72470 | A | A | A | T | T |
| SNP | 72581 | C | C | C | T | T |
| SNP | 72584 | T | T | T | C | C |
| SNP | 72594 | G | G | G | A | A |
| SNP | 72617 | C | C | C | G | G |
| SNP | 72686 | G | G | G | T | T |
| SNP | 72729 | C | C | C | A | A |
| SNP | 72793 | T | T | T | C | C |
| SNP | 72951 | G | T | T | G | G |
| SNP | 73052 | A | A | A | T | T |
| SNP | 73060 | T | T | T | A | A |
| SNP | 73070 | T | T | T | G | G |
| SNP | 73926 | C | C | C | T | T |
| SNP | 74099 | T | T | T | G | G |
| SNP | 74419 | G | G | G | A | A |
| SNP | 75062 | A | A | A | G | G |
| SNP | 75176 | C | C | C | T | T |
| SNP | 75729 | T | T | T | C | C |
| SNP | 77025 | A | A | A | C | C |
| SNP | 77334 | T | T | T | C | C |
| SNP | 77406 | G | T | T | T | T |
| SNP | 77574 | A | A | A | C | C |
| SNP | 77959 | G | G | G | T | T |
| SNP | 77971 | G | G | G | A | A |
| SNP | 78172 | G | G | G | T | T |
| SNP | 78205 | G | G | G | A | A |
| SNP | 78811 | T | C | C | C | C |
| SNP | 79839 | C | C | C | T | T |
| SNP | 79844 | A | A | A | C | C |
| SNP | 79848 | G | T | T | T | T |
| SNP | 79909 | C | C | C | A | A |
| SNP | 80704 | C | C | C | T | T |
| SNP | 80727 | A | A | A | G | G |
| SNP | 80828 | A | A | A | G | G |
| SNP | 80864 | A | A | A | G | G |
| SNP | 81031 | C | C | C | T | T |
| SNP | 81424 | T | T | T | G | G |
| SNP | 81499 | C | C | C | T | T |
| SNP | 81584 | A | A | A | G | G |
| SNP | 82351 | G | A | A | G | G |
| SNP | 82555 | G | C | C | C | C |
| SNP | 82748 | C | C | C | T | T |
| SNP | 82870 | A | T | T | A | A |
| SNP | 83324 | T | T | T | C | C |
| SNP | 83878 | C | A | A | C | C |
| SNP | 83880 | T | A | A | T | T |
| SNP | 84088 | T | T | T | G | G |
| SNP | 84673 | G | G | G | A | A |
| SNP | 84686 | C | C | C | A | A |
| SNP | 84724 | C | C | C | A | A |
| SNP | 84855 | G | A | A | G | G |
| SNP | 84968 | T | T | T | C | C |
| SNP | 85374 | C | G | G | C | C |
| SNP | 85530 | T | T | T | C | C |
| SNP | 85723 | T | G | G | T | T |
| SNP | 85853 | C | C | C | T | T |
| SNP | 86023 | A | A | A | G | G |
| SNP | 86718 | C | C | C | G | G |
| SNP | 86941 | T | C | C | C | C |
| SNP | 87353 | G | G | G | A | A |
| SNP | 87370 | C | C | C | T | T |
| SNP | 87923 | T | T | T | C | C |
| SNP | 88323 | C | T | T | C | C |
| SNP | 88504 | C | C | C | T | T |
| SNP | 88657 | C | C | C | T | T |
| SNP | 88941 | A | A | A | C | C |
| SNP | 88983 | C | C | C | A | A |
| SNP | 89028 | G | T | T | G | G |
| SNP | 89187 | T | C | C | C | C |
| SNP | 89199 | G | G | G | T | T |
| SNP | 89440 | T | T | T | C | C |
| SNP | 89474 | T | T | T | G | G |
| SNP | 89503 | G | G | G | T | T |
| SNP | 89608 | C | C | C | T | T |
| SNP | 89862 | G | G | G | A | A |
| SNP | 89966 | T | G | G | T | T |
| SNP | 90175 | A | C | C | C | C |
| SNP | 95903 | G | G | G | A | A |
| SNP | 98854 | C | C | C | T | T |
| SNP | 103390 | G | G | G | T | T |
| SNP | 104558 | T | T | T | G | G |
| SNP | 104601 | G | G | G | T | T |
| SNP | 107888 | G | T | T | G | G |
| SNP | 109106 | A | G | G | A | A |
| SNP | 111837 | A | A | A | G | G |
| SNP | 113773 | C | C | C | T | T |
| SNP | 114147 | G | G | G | T | T |
| SNP | 114310 | G | G | G | A | A |
| SNP | 114583 | C | A | A | A | A |
| SNP | 114584 | T | A | A | A | A |
| SNP | 114585 | T | A | A | A | A |
| SNP | 114586 | T | G | G | G | G |
| SNP | 115067 | T | T | T | C | C |
| SNP | 115263 | C | C | C | T | T |
| SNP | 115564 | C | C | C | T | T |
| SNP | 116152 | T | T | T | G | G |
| SNP | 116153 | T | T | T | A | A |
| SNP | 116158 | A | A | A | A | G |
| SNP | 116191 | T | C | C | T | T |
| SNP | 116253 | C | C | C | T | T |
| SNP | 116692 | C | C | C | A | A |
| SNP | 116964 | G | T | T | G | G |
| SNP | 117298 | A | A | A | C | C |
| SNP | 117494 | C | T | T | C | C |
| SNP | 117608 | C | C | C | T | T |
| SNP | 117823 | T | T | T | G | G |
| SNP | 118561 | G | G | G | A | A |
| SNP | 118639 | C | C | C | G | G |
| SNP | 118694 | C | A | A | C | C |
| SNP | 118748 | C | C | C | G | G |
| SNP | 118793 | C | C | C | T | T |
| SNP | 118814 | A | A | A | C | C |
| SNP | 118815 | G | G | G | C | C |
| SNP | 119045 | A | A | A | T | T |
| SNP | 119075 | A | A | A | C | C |
| SNP | 119170 | A | A | A | C | C |
| SNP | 119359 | T | T | T | G | G |
| SNP | 119579 | T | T | A | T | T |
| SNP | 119583 | T | T | A | T | T |
| SNP | 119689 | C | C | C | A | A |
| SNP | 119723 | A | A | A | C | C |
| SNP | 119745 | A | A | A | G | G |
| SNP | 119850 | T | T | T | G | G |
| SNP | 119861 | A | A | A | C | C |
| SNP | 119949 | G | G | G | C | C |
| SNP | 120030 | A | G | G | G | G |
| SNP | 120153 | G | G | G | A | A |
| SNP | 120165 | A | A | A | C | C |
| SNP | 120267 | T | T | T | A | A |
| SNP | 120280 | T | T | T | C | C |
| SNP | 120313 | T | T | T | C | C |
| SNP | 120459 | G | A | A | G | G |
| SNP | 120894 | C | T | T | C | C |
| SNP | 120900 | G | G | G | A | A |
| SNP | 121277 | C | C | C | T | T |
| SNP | 121383 | T | T | T | G | G |
| SNP | 121460 | T | T | T | C | C |
| SNP | 121523 | A | A | A | T | A |
| SNP | 121925 | A | C | C | A | A |
| SNP | 122036 | G | G | G | C | C |
| SNP | 122096 | G | G | G | A | A |
| SNP | 122104 | T | T | T | C | C |
| SNP | 122114 | A | G | G | G | G |
| SNP | 122126 | C | T | T | T | T |
| SNP | 122243 | G | G | G | A | A |
| SNP | 122381 | G | G | G | A | A |
| SNP | 123006 | G | G | G | A | A |
| SNP | 123067 | A | A | A | T | T |
| SNP | 123103 | T | T | T | C | C |
| SNP | 123200 | G | G | G | A | A |
| SNP | 123232 | C | C | C | A | A |
| SNP | 124161 | G | G | G | A | A |
| SNP | 124162 | G | G | G | A | A |
| SNP | 124250 | G | G | G | A | A |
| SNP | 124260 | G | G | G | A | A |
| SNP | 124288 | G | G | G | T | T |
| SNP | 124540 | C | C | C | T | T |
| SNP | 124909 | C | T | T | T | T |
| SNP | 125099 | C | T | T | T | T |
| SNP | 125104 | C | C | C | A | A |
| SNP | 125198 | T | T | T | C | C |
| SNP | 125798 | G | G | G | T | T |
| SNP | 126283 | T | T | T | C | C |
| SNP | 126296 | C | C | C | A | A |
| SNP | 126297 | A | C | C | A | A |
| SNP | 126438 | G | C | C | C | C |
| SNP | 126811 | T | T | T | A | A |
| SNP | 126815 | G | G | G | A | A |
| SNP | 126817 | G | G | G | A | A |
| SNP | 126818 | A | A | A | G | G |
| SNP | 126819 | T | T | T | A | A |
| SNP | 126820 | C | C | C | T | T |
| SNP | 126821 | T | T | T | C | C |
| SNP | 126823 | T | T | T | C | C |
| SNP | 126895 | A | G | G | G | G |
| SNP | 127142 | C | C | C | T | T |
| SNP | 127181 | T | T | T | G | G |
| SNP | 127222 | C | C | C | T | T |
| SNP | 127477 | G | G | G | A | A |
| SNP | 127486 | A | A | A | G | G |
| SNP | 127853 | C | C | C | T | T |
| SNP | 127982 | T | T | T | C | C |
| SNP | 128016 | C | C | C | T | T |
| SNP | 128366 | G | G | G | A | A |
| SNP | 128524 | G | G | G | A | A |
| SNP | 128712 | C | C | C | T | T |
| SNP | 128758 | T | C | C | C | C |
| SNP | 128995 | T | T | T | C | C |
| SNP | 129358 | G | G | G | T | T |
| SNP | 129681 | A | C | C | C | C |
| SNP | 129761 | A | A | A | G | G |
| SNP | 129877 | T | T | T | A | A |
| SNP | 129940 | T | T | T | A | A |
| SNP | 130082 | C | C | C | A | A |
| SNP | 130272 | T | T | T | G | G |
| SNP | 130338 | G | G | G | A | A |
| SNP | 130395 | A | A | A | C | C |
| SNP | 130464 | C | C | C | C | A |
| SNP | 130806 | A | A | A | C | C |
| SNP | 130912 | C | T | T | C | C |
| SNP | 131013 | G | G | G | C | C |
| SNP | 131027 | G | T | T | G | G |
| SNP | 131029 | G | T | T | G | G |
| SNP | 131030 | A | C | C | A | A |
| SNP | 131032 | A | C | C | A | A |
| SNP | 131040 | A | A | A | C | C |
| SNP | 131116 | A | A | A | G | G |
| SNP | 131197 | C | T | T | C | C |
| SNP | 131417 | T | T | T | A | A |
| SNP | 131449 | G | G | G | C | C |
| SNP | 131714 | C | C | C | A | A |
| SNP | 131715 | C | C | C | T | T |
| SNP | 132094 | C | C | C | T | T |
| SNP | 132122 | C | C | C | A | A |
| SNP | 132234 | G | G | G | A | A |
| SNP | 132253 | A | A | A | T | T |
| SNP | 132254 | A | T | T | T | T |
| SNP | 132258 | T | T | T | A | A |
| SNP | 132364 | G | G | G | A | A |
| SNP | 132528 | A | A | A | G | G |
| SNP | 132551 | T | T | T | G | G |
| SNP | 132587 | T | T | T | C | C |
| SNP | 132621 | A | A | A | G | G |
| SNP | 132650 | A | C | C | C | C |
| SNP | 132656 | T | T | T | C | C |
| SNP | 132700 | C | C | C | A | A |
| SNP | 132743 | T | T | T | G | G |
| SNP | 132821 | T | T | T | A | T |
| SNP | 132907 | A | A | A | T | T |
| SNP | 133012 | C | C | C | T | T |
| SNP | 133125 | T | C | C | C | C |
| SNP | 133406 | C | C | C | T | T |
| SNP | 133565 | G | G | G | C | C |
| SNP | 133580 | T | C | C | C | C |
| SNP | 133961 | T | T | T | A | A |
| SNP | 133988 | T | G | G | T | T |
| SNP | 134065 | A | A | A | C | C |
| SNP | 134132 | G | G | G | A | A |
| SNP | 134175 | C | C | C | A | A |
| SNP | 134184 | A | A | A | C | C |
| SNP | 134415 | A | A | A | G | G |
| SNP | 134476 | G | T | T | G | G |
| SNP | 134493 | G | A | A | A | A |
| SNP | 134582 | T | T | T | G | G |
| SNP | 134612 | T | T | T | T | C |
| SNP | 134618 | T | T | T | C | C |
| SNP | 134621 | C | C | C | T | T |
| SNP | 134622 | A | A | A | T | T |
| SNP | 134623 | G | G | G | T | T |
| SNP | 134624 | C | C | C | T | T |
| SNP | 135199 | G | G | G | A | A |
| SNP | 135500 | G | G | G | A | A |
| SNP | 135696 | A | A | A | G | G |
| SNP | 136177 | A | C | C | C | C |
| SNP | 136178 | A | T | T | T | T |
| SNP | 136179 | A | T | T | T | T |
| SNP | 136180 | G | T | T | T | T |
| SNP | 136453 | C | C | C | T | T |
| SNP | 136616 | C | C | C | A | A |
| SNP | 136990 | G | G | G | A | A |
| SNP | 138926 | T | T | T | C | C |
| SNP | 141657 | T | C | C | T | T |
| SNP | 142875 | C | A | A | C | C |
| SNP | 146162 | C | C | C | A | A |
| SNP | 146205 | A | A | A | C | C |
| SNP | 147373 | C | C | C | A | A |
| SNP | 151909 | G | G | G | A | A |
| SNP | 154860 | C | C | C | T | T |
| SNP | 160390 | G | G | A | G | G |
| SNP | 160391 | T | T | A | T | T |
| SNP | 160419 | C | C | A | C | C |
| SNP | 160421 | T | T | C | T | T |
| SNP | 160422 | G | G | A | G | G |
| SNP | 160588 | T | G | G | G | G |
| Type | position | *J. cathayensis* | *J. hopeiensis* | *J. mandshurica* | *J. regia* | *J. sigillata* |
| Indel | 1 | G | - | - | A | A |
| Indel | 2 | G | - | - | A | A |
| Indel | 3 | G | - | - | A | A |
| Indel | 4 | G | - | - | A | A |
| Indel | 5 | G | - | - | A | A |
| Indel | 6 | G | - | - | A | A |
| Indel | 7 | A | G | G | - | - |
| Indel | 8 | A | G | G | - | - |
| Indel | 9 | A | G | G | - | - |
| Indel | 10 | A | G | G | - | - |
| Indel | 11 | A | G | G | - | - |
| Indel | 1553 | G | - | - | A | A |
| Indel | 3530 | G | - | - | A | A |
| Indel | 3733 | A | G | G | G | G |
| Indel | 3734 | A | G | G | G | G |
| Indel | 4012 | A | - | - | G | G |
| Indel | 4013 | A | - | - | G | G |
| Indel | 4507 | A | G | G | - | - |
| Indel | 4571 | G | - | - | A | A |
| Indel | 4572 | G | - | - | A | A |
| Indel | 4573 | G | A | A | A | A |
| Indel | 4800 | G | A | A | - | - |
| Indel | 4989 | G | - | - | A | A |
| Indel | 4990 | G | - | - | A | A |
| Indel | 5102 | A | G | G | G | G |
| Indel | 5103 | A | - | - | G | G |
| Indel | 5104 | A | - | - | G | G |
| Indel | 5105 | A | - | - | G | G |
| Indel | 5106 | A | - | - | G | G |
| Indel | 5107 | A | - | - | G | G |
| Indel | 5108 | A | - | - | G | G |
| Indel | 5109 | A | - | - | G | G |
| Indel | 5110 | A | - | - | G | G |
| Indel | 5111 | A | - | - | G | G |
| Indel | 5112 | A | - | - | G | G |
| Indel | 5113 | A | - | - | G | G |
| Indel | 5114 | A | - | - | G | G |
| Indel | 5115 | A | - | - | G | G |
| Indel | 5116 | A | - | - | G | G |
| Indel | 5117 | A | - | - | G | G |
| Indel | 5118 | A | - | - | G | G |
| Indel | 5119 | A | - | - | G | G |
| Indel | 5120 | A | - | - | G | G |
| Indel | 5121 | A | - | - | G | G |
| Indel | 5122 | A | - | - | G | G |
| Indel | 5123 | A | - | - | G | G |
| Indel | 5124 | A | - | - | G | G |
| Indel | 5125 | A | - | - | G | G |
| Indel | 5126 | A | - | - | G | G |
| Indel | 5316 | G | - | - | A | A |
| Indel | 5317 | G | - | - | A | A |
| Indel | 6705 | G | - | - | A | A |
| Indel | 6723 | G | - | - | A | A |
| Indel | 6724 | G | - | - | A | A |
| Indel | 6725 | G | - | - | A | A |
| Indel | 6726 | G | - | - | A | A |
| Indel | 6727 | G | - | - | A | A |
| Indel | 6728 | G | - | - | A | A |
| Indel | 6729 | G | - | - | A | A |
| Indel | 6837 | G | - | - | A | A |
| Indel | 6838 | G | - | - | A | A |
| Indel | 6839 | G | - | - | A | A |
| Indel | 6840 | G | - | - | A | A |
| Indel | 6841 | G | - | - | A | A |
| Indel | 6842 | G | - | - | A | A |
| Indel | 6843 | G | - | - | A | A |
| Indel | 6844 | G | - | - | A | A |
| Indel | 6845 | G | - | - | A | A |
| Indel | 6846 | G | - | - | A | A |
| Indel | 6847 | G | - | - | A | A |
| Indel | 6848 | G | - | - | A | A |
| Indel | 6849 | G | - | - | A | A |
| Indel | 6968 | A | G | G | G | G |
| Indel | 6987 | G | - | - | A | A |
| Indel | 7574 | A | G | G | - | - |
| Indel | 7731 | A | - | - | G | G |
| Indel | 7732 | A | - | - | G | G |
| Indel | 7733 | A | - | - | G | G |
| Indel | 7734 | A | - | - | G | G |
| Indel | 7735 | A | - | - | G | G |
| Indel | 7736 | A | - | - | G | G |
| Indel | 7737 | A | - | - | G | G |
| Indel | 7738 | A | - | - | G | G |
| Indel | 7739 | A | - | - | G | G |
| Indel | 7740 | A | - | - | G | G |
| Indel | 7741 | A | - | - | G | G |
| Indel | 7837 | A | - | - | G | G |
| Indel | 8334 | G | - | - | A | A |
| Indel | 8335 | G | - | - | A | A |
| Indel | 8336 | G | - | - | A | A |
| Indel | 8337 | G | - | - | A | A |
| Indel | 8338 | G | - | - | A | A |
| Indel | 8339 | G | - | - | A | A |
| Indel | 8402 | G | A | A | - | - |
| Indel | 8403 | G | A | A | - | - |
| Indel | 8404 | A | - | - | G | G |
| Indel | 8928 | G | - | - | A | A |
| Indel | 9071 | G | - | - | A | - |
| Indel | 9072 | G | - | - | A | A |
| Indel | 9073 | G | A | A | A | A |
| Indel | 9340 | G | - | - | A | A |
| Indel | 9537 | G | - | - | A | A |
| Indel | 9538 | G | - | - | A | A |
| Indel | 9539 | G | - | - | A | A |
| Indel | 9540 | G | - | - | A | A |
| Indel | 9541 | G | - | - | A | A |
| Indel | 9542 | G | - | - | A | A |
| Indel | 9543 | G | - | - | A | A |
| Indel | 9544 | G | - | - | A | A |
| Indel | 9545 | G | - | - | A | A |
| Indel | 9546 | G | - | - | A | A |
| Indel | 9547 | G | - | - | A | A |
| Indel | 9548 | G | - | - | A | A |
| Indel | 9549 | G | - | - | A | A |
| Indel | 9550 | G | - | - | A | A |
| Indel | 9551 | G | - | - | A | A |
| Indel | 9552 | G | - | - | A | A |
| Indel | 9553 | G | - | - | A | A |
| Indel | 9554 | G | - | - | A | A |
| Indel | 9555 | G | - | - | A | A |
| Indel | 9556 | G | - | - | A | A |
| Indel | 9557 | G | - | - | A | A |
| Indel | 9558 | G | - | - | A | A |
| Indel | 9559 | G | - | - | A | A |
| Indel | 9609 | A | - | - | G | G |
| Indel | 9610 | A | - | - | G | G |
| Indel | 9611 | A | - | - | G | G |
| Indel | 9612 | A | - | - | G | G |
| Indel | 9613 | A | - | - | G | G |
| Indel | 9614 | A | - | - | G | G |
| Indel | 9615 | A | - | - | G | G |
| Indel | 9616 | A | - | - | G | G |
| Indel | 9617 | A | - | - | G | G |
| Indel | 9618 | A | - | - | G | G |
| Indel | 9619 | A | - | - | G | G |
| Indel | 9620 | A | - | - | G | G |
| Indel | 10033 | G | A | A | A | A |
| Indel | 10338 | A | G | G | - | - |
| Indel | 10500 | G | A | A | - | - |
| Indel | 10501 | A | - | - | G | G |
| Indel | 11152 | G | - | - | A | A |
| Indel | 11153 | G | - | - | A | A |
| Indel | 11154 | G | - | - | A | A |
| Indel | 11155 | G | - | - | A | A |
| Indel | 11156 | G | - | - | A | A |
| Indel | 11157 | G | - | - | A | A |
| Indel | 11158 | G | - | - | A | A |
| Indel | 11409 | A | - | - | G | G |
| Indel | 13325 | G | - | - | A | A |
| Indel | 13956 | G | A | A | - | - |
| Indel | 14048 | A | - | - | G | G |
| Indel | 14480 | G | A | A | - | - |
| Indel | 14852 | A | G | G | G | G |
| Indel | 14882 | G | A | A | - | - |
| Indel | 14883 | A | - | - | G | G |
| Indel | 14988 | G | - | - | A | A |
| Indel | 14989 | G | - | - | A | A |
| Indel | 14990 | G | - | - | A | A |
| Indel | 14991 | G | - | - | A | A |
| Indel | 14992 | G | - | - | A | A |
| Indel | 15680 | G | - | - | A | A |
| Indel | 16017 | G | A | A | - | - |
| Indel | 16018 | G | A | A | - | - |
| Indel | 16039 | G | - | - | A | A |
| Indel | 16040 | G | - | - | A | A |
| Indel | 16041 | G | - | - | A | A |
| Indel | 16478 | A | - | - | G | G |
| Indel | 16479 | A | - | - | G | G |
| Indel | 16480 | A | - | - | G | G |
| Indel | 16481 | A | - | - | G | G |
| Indel | 16482 | A | - | - | G | G |
| Indel | 16483 | A | - | - | G | G |
| Indel | 16484 | A | - | - | G | G |
| Indel | 16485 | A | - | - | G | G |
| Indel | 16486 | A | - | - | G | G |
| Indel | 17465 | G | - | - | A | A |
| Indel | 18408 | A | - | - | G | G |
| Indel | 18409 | G | A | A | - | - |
| Indel | 18733 | G | A | A | A | A |
| Indel | 18734 | G | A | A | - | - |
| Indel | 19603 | G | - | - | A | A |
| Indel | 19604 | G | - | - | A | A |
| Indel | 19605 | G | - | - | A | A |
| Indel | 19606 | G | - | - | A | A |
| Indel | 19607 | G | - | - | A | A |
| Indel | 19608 | G | - | - | A | A |
| Indel | 19609 | G | - | - | A | A |
| Indel | 19610 | G | - | - | A | A |
| Indel | 19611 | G | - | - | A | A |
| Indel | 19612 | G | - | - | A | A |
| Indel | 19613 | G | - | - | A | A |
| Indel | 19614 | G | - | - | A | A |
| Indel | 19615 | G | - | - | A | A |
| Indel | 19616 | G | - | - | A | A |
| Indel | 19617 | G | - | - | A | A |
| Indel | 24794 | G | A | A | - | - |
| Indel | 24872 | A | - | - | G | G |
| Indel | 24873 | G | A | A | - | - |
| Indel | 25220 | G | - | - | A | A |
| Indel | 25221 | G | - | - | A | A |
| Indel | 25222 | G | - | - | A | A |
| Indel | 25223 | G | - | - | A | A |
| Indel | 25224 | G | - | - | A | A |
| Indel | 30311 | A | - | - | G | G |
| Indel | 30437 | A | - | - | G | G |
| Indel | 30898 | A | G | G | - | - |
| Indel | 30899 | A | G | G | - | - |
| Indel | 30900 | A | G | G | - | - |
| Indel | 30901 | A | G | G | - | - |
| Indel | 30902 | A | G | G | - | - |
| Indel | 30903 | A | G | G | - | - |
| Indel | 30904 | A | G | G | - | - |
| Indel | 30905 | A | G | G | - | - |
| Indel | 30906 | A | G | G | - | - |
| Indel | 31503 | G | - | - | A | A |
| Indel | 31504 | G | - | - | A | A |
| Indel | 31505 | G | - | - | A | A |
| Indel | 31506 | G | - | - | A | A |
| Indel | 31507 | G | - | - | A | A |
| Indel | 31508 | G | - | - | A | A |
| Indel | 31509 | G | - | - | A | A |
| Indel | 31510 | G | - | - | A | A |
| Indel | 31511 | G | - | - | A | A |
| Indel | 31512 | G | - | - | A | A |
| Indel | 31513 | G | - | - | A | A |
| Indel | 31514 | G | - | - | A | A |
| Indel | 31515 | G | - | - | A | A |
| Indel | 31516 | G | - | - | A | A |
| Indel | 31517 | G | - | - | A | A |
| Indel | 31518 | G | - | - | A | A |
| Indel | 31519 | G | - | - | A | A |
| Indel | 31520 | G | - | - | A | A |
| Indel | 31521 | G | - | - | A | A |
| Indel | 31522 | G | - | - | A | A |
| Indel | 31523 | G | - | - | A | A |
| Indel | 31524 | G | - | - | A | A |
| Indel | 31525 | G | - | - | A | A |
| Indel | 31526 | G | - | - | A | A |
| Indel | 31527 | G | - | - | A | A |
| Indel | 31528 | G | - | - | A | A |
| Indel | 31529 | G | - | - | A | A |
| Indel | 31530 | G | - | - | A | A |
| Indel | 31531 | G | - | - | A | A |
| Indel | 31532 | G | - | - | A | A |
| Indel | 31533 | G | - | - | A | A |
| Indel | 31534 | G | - | - | A | A |
| Indel | 31535 | G | - | - | A | A |
| Indel | 31536 | G | - | - | A | A |
| Indel | 31537 | G | - | - | A | A |
| Indel | 31538 | G | - | - | A | A |
| Indel | 31539 | G | - | - | A | A |
| Indel | 31540 | G | - | - | A | A |
| Indel | 31541 | G | - | - | A | A |
| Indel | 31542 | G | - | - | A | A |
| Indel | 31543 | G | - | - | A | A |
| Indel | 31544 | G | - | - | A | A |
| Indel | 31545 | G | - | - | A | A |
| Indel | 31546 | G | - | - | A | A |
| Indel | 31547 | G | - | - | A | A |
| Indel | 31548 | G | - | - | A | A |
| Indel | 31549 | G | - | - | A | A |
| Indel | 31550 | G | - | - | A | A |
| Indel | 31551 | G | - | - | A | A |
| Indel | 31552 | G | - | - | A | A |
| Indel | 31553 | G | - | - | A | A |
| Indel | 31554 | G | - | - | A | A |
| Indel | 31555 | G | - | - | A | A |
| Indel | 31556 | G | - | - | A | A |
| Indel | 31557 | G | - | - | A | A |
| Indel | 31558 | G | - | - | A | A |
| Indel | 31559 | G | - | - | A | A |
| Indel | 31560 | G | - | - | A | A |
| Indel | 31561 | G | - | - | A | A |
| Indel | 31562 | G | - | - | A | A |
| Indel | 31563 | G | - | - | A | A |
| Indel | 31564 | G | - | - | A | A |
| Indel | 31565 | G | - | - | A | A |
| Indel | 31566 | G | - | - | A | A |
| Indel | 31567 | G | - | - | A | A |
| Indel | 31568 | G | - | - | A | A |
| Indel | 31569 | G | - | - | A | A |
| Indel | 31570 | G | - | - | A | A |
| Indel | 31571 | G | - | - | A | A |
| Indel | 31572 | G | - | - | A | A |
| Indel | 31573 | G | - | - | A | A |
| Indel | 31574 | G | - | - | A | A |
| Indel | 31575 | G | - | - | A | A |
| Indel | 31576 | G | - | - | A | A |
| Indel | 31577 | G | - | - | A | A |
| Indel | 31578 | G | - | - | A | A |
| Indel | 31579 | G | - | - | A | A |
| Indel | 31580 | G | - | - | A | A |
| Indel | 31581 | G | - | - | A | A |
| Indel | 31582 | G | - | - | A | A |
| Indel | 31583 | G | - | - | A | A |
| Indel | 31584 | G | - | - | A | A |
| Indel | 31585 | G | - | - | A | A |
| Indel | 31586 | G | - | - | A | A |
| Indel | 31587 | G | - | - | A | A |
| Indel | 31588 | G | - | - | A | A |
| Indel | 31589 | G | - | - | A | A |
| Indel | 31590 | G | - | - | A | A |
| Indel | 31591 | G | - | - | A | A |
| Indel | 31592 | G | - | - | A | A |
| Indel | 31593 | G | - | - | A | A |
| Indel | 31594 | G | - | - | A | A |
| Indel | 31595 | G | - | - | A | A |
| Indel | 31596 | G | - | - | A | A |
| Indel | 31597 | G | - | - | A | A |
| Indel | 31598 | G | - | - | A | A |
| Indel | 31599 | G | - | - | A | A |
| Indel | 31600 | G | - | - | A | A |
| Indel | 31601 | G | - | - | A | A |
| Indel | 31602 | G | - | - | A | A |
| Indel | 31603 | G | - | - | A | A |
| Indel | 31604 | G | - | - | A | A |
| Indel | 31605 | G | - | - | A | A |
| Indel | 31606 | G | - | - | A | A |
| Indel | 31607 | G | - | - | A | A |
| Indel | 31608 | G | - | - | A | A |
| Indel | 31609 | G | - | - | A | A |
| Indel | 31610 | G | - | - | A | A |
| Indel | 31611 | G | - | - | A | A |
| Indel | 31612 | G | - | - | A | A |
| Indel | 31613 | G | - | - | A | A |
| Indel | 31614 | G | - | - | A | A |
| Indel | 31615 | G | - | - | A | A |
| Indel | 31616 | G | - | - | A | A |
| Indel | 31617 | G | - | - | A | A |
| Indel | 31618 | G | - | - | A | A |
| Indel | 31619 | G | - | - | A | A |
| Indel | 31620 | G | - | - | A | A |
| Indel | 31621 | G | - | - | A | A |
| Indel | 31622 | G | - | - | A | A |
| Indel | 31623 | G | - | - | A | A |
| Indel | 31624 | G | - | - | A | A |
| Indel | 31625 | G | - | - | A | A |
| Indel | 31626 | G | - | - | A | A |
| Indel | 31627 | G | - | - | A | A |
| Indel | 31628 | G | - | - | A | A |
| Indel | 31629 | G | - | - | A | A |
| Indel | 31630 | G | - | - | A | A |
| Indel | 31631 | G | - | - | A | A |
| Indel | 31632 | G | - | - | A | A |
| Indel | 31633 | G | - | - | A | A |
| Indel | 31634 | G | - | - | A | A |
| Indel | 31635 | G | - | - | A | A |
| Indel | 31636 | G | - | - | A | A |
| Indel | 31637 | G | - | - | A | A |
| Indel | 31638 | G | - | - | A | A |
| Indel | 31639 | G | - | - | A | A |
| Indel | 31640 | G | - | - | A | A |
| Indel | 31641 | G | - | - | A | A |
| Indel | 31642 | G | - | - | A | A |
| Indel | 31643 | G | - | - | A | A |
| Indel | 31644 | G | - | - | A | A |
| Indel | 31645 | G | - | - | A | A |
| Indel | 31646 | G | - | - | A | A |
| Indel | 31647 | G | - | - | A | A |
| Indel | 31648 | G | - | - | A | A |
| Indel | 31649 | G | - | - | A | A |
| Indel | 31650 | G | - | - | A | A |
| Indel | 31651 | G | - | - | A | A |
| Indel | 31652 | G | - | - | A | A |
| Indel | 31653 | G | - | - | A | A |
| Indel | 31654 | G | - | - | A | A |
| Indel | 31655 | G | - | - | A | A |
| Indel | 31656 | G | - | - | A | A |
| Indel | 31657 | G | - | - | A | A |
| Indel | 31658 | G | - | - | A | A |
| Indel | 31659 | G | - | - | A | A |
| Indel | 31660 | G | - | - | A | A |
| Indel | 31661 | G | - | - | A | A |
| Indel | 31662 | G | - | - | A | A |
| Indel | 31663 | G | - | - | A | A |
| Indel | 31664 | G | - | - | A | A |
| Indel | 31665 | G | - | - | A | A |
| Indel | 31666 | G | - | - | A | A |
| Indel | 31667 | G | - | - | A | A |
| Indel | 31668 | G | - | - | A | A |
| Indel | 31669 | G | - | - | A | A |
| Indel | 31670 | G | - | - | A | A |
| Indel | 31671 | G | - | - | A | A |
| Indel | 31672 | G | - | - | A | A |
| Indel | 31673 | G | - | - | A | A |
| Indel | 31674 | G | - | - | A | A |
| Indel | 31675 | G | - | - | A | A |
| Indel | 31676 | G | - | - | A | A |
| Indel | 31677 | G | - | - | A | A |
| Indel | 31678 | G | - | - | A | A |
| Indel | 31679 | G | - | - | A | A |
| Indel | 31680 | G | - | - | A | A |
| Indel | 31681 | G | - | - | A | A |
| Indel | 31682 | G | - | - | A | A |
| Indel | 31683 | G | - | - | A | A |
| Indel | 31684 | G | - | - | A | A |
| Indel | 31685 | G | - | - | A | A |
| Indel | 31686 | G | - | - | A | A |
| Indel | 31687 | G | - | - | A | A |
| Indel | 31688 | G | - | - | A | A |
| Indel | 31689 | G | - | - | A | A |
| Indel | 31690 | G | - | - | A | A |
| Indel | 31691 | G | - | - | A | A |
| Indel | 31692 | G | - | - | A | A |
| Indel | 31693 | G | - | - | A | A |
| Indel | 31694 | G | - | - | A | A |
| Indel | 31695 | G | - | - | A | A |
| Indel | 31696 | G | - | - | A | A |
| Indel | 31697 | G | - | - | A | A |
| Indel | 31698 | G | - | - | A | A |
| Indel | 31699 | G | - | - | A | A |
| Indel | 31700 | G | - | - | A | A |
| Indel | 31701 | G | - | - | A | A |
| Indel | 31702 | G | - | - | A | A |
| Indel | 31703 | G | - | - | A | A |
| Indel | 31704 | G | - | - | A | A |
| Indel | 31705 | G | - | - | A | A |
| Indel | 31706 | G | - | - | A | A |
| Indel | 31707 | G | - | - | A | A |
| Indel | 31708 | G | - | - | A | A |
| Indel | 31709 | G | - | - | A | A |
| Indel | 31710 | G | - | - | A | A |
| Indel | 31711 | G | - | - | A | A |
| Indel | 31712 | G | - | - | A | A |
| Indel | 31713 | G | - | - | A | A |
| Indel | 31714 | G | - | - | A | A |
| Indel | 31715 | G | - | - | A | A |
| Indel | 31716 | G | - | - | A | A |
| Indel | 31717 | G | - | - | A | A |
| Indel | 31718 | G | - | - | A | A |
| Indel | 31719 | G | - | - | A | A |
| Indel | 31720 | G | - | - | A | A |
| Indel | 31721 | G | - | - | A | A |
| Indel | 31722 | G | - | - | A | A |
| Indel | 31723 | G | - | - | A | A |
| Indel | 31724 | G | - | - | A | A |
| Indel | 31725 | G | - | - | A | A |
| Indel | 31726 | G | - | - | A | A |
| Indel | 31727 | G | - | - | A | A |
| Indel | 31728 | G | - | - | A | A |
| Indel | 31729 | G | - | - | A | A |
| Indel | 31730 | G | - | - | A | A |
| Indel | 31731 | G | - | - | A | A |
| Indel | 31732 | G | - | - | A | A |
| Indel | 31733 | G | - | - | A | A |
| Indel | 31734 | G | - | - | A | A |
| Indel | 31735 | G | - | - | A | A |
| Indel | 31736 | G | - | - | A | A |
| Indel | 31737 | G | - | - | A | A |
| Indel | 31738 | G | - | - | A | A |
| Indel | 31739 | G | - | - | A | A |
| Indel | 31740 | G | - | - | A | A |
| Indel | 31741 | G | - | - | A | A |
| Indel | 31742 | G | - | - | A | A |
| Indel | 31743 | G | - | - | A | A |
| Indel | 31744 | G | - | - | A | A |
| Indel | 31745 | G | - | - | A | A |
| Indel | 31746 | G | - | - | A | A |
| Indel | 31747 | G | - | - | A | A |
| Indel | 31748 | G | - | - | A | A |
| Indel | 31749 | G | - | - | A | A |
| Indel | 31750 | G | - | - | A | A |
| Indel | 31751 | G | - | - | A | A |
| Indel | 31752 | G | - | - | A | A |
| Indel | 31753 | G | - | - | A | A |
| Indel | 31754 | G | - | - | A | A |
| Indel | 31755 | G | - | - | A | A |
| Indel | 31756 | G | - | - | A | A |
| Indel | 31757 | G | - | - | A | A |
| Indel | 31758 | G | - | - | A | A |
| Indel | 31759 | G | - | - | A | A |
| Indel | 31760 | G | - | - | A | A |
| Indel | 31761 | G | - | - | A | A |
| Indel | 31762 | G | - | - | A | A |
| Indel | 31763 | G | - | - | A | A |
| Indel | 31764 | G | - | - | A | A |
| Indel | 31765 | G | - | - | A | A |
| Indel | 31766 | G | - | - | A | A |
| Indel | 31767 | G | - | - | A | A |
| Indel | 31768 | G | - | - | A | A |
| Indel | 31769 | G | - | - | A | A |
| Indel | 31770 | G | - | - | A | A |
| Indel | 31771 | G | - | - | A | A |
| Indel | 31772 | G | - | - | A | A |
| Indel | 31773 | G | - | - | A | A |
| Indel | 31774 | G | - | - | A | A |
| Indel | 31775 | G | - | - | A | A |
| Indel | 31776 | G | - | - | A | A |
| Indel | 31777 | G | - | - | A | A |
| Indel | 31778 | G | - | - | A | A |
| Indel | 31779 | G | - | - | A | A |
| Indel | 31780 | G | - | - | A | A |
| Indel | 31781 | G | - | - | A | A |
| Indel | 31782 | G | - | - | A | A |
| Indel | 31783 | G | - | - | A | A |
| Indel | 31784 | G | - | - | A | A |
| Indel | 31785 | G | - | - | A | A |
| Indel | 31786 | G | - | - | A | A |
| Indel | 31787 | G | - | - | A | A |
| Indel | 31788 | G | - | - | A | A |
| Indel | 31789 | G | - | - | A | A |
| Indel | 31790 | G | - | - | A | A |
| Indel | 31791 | G | - | - | A | A |
| Indel | 31792 | G | - | - | A | A |
| Indel | 31793 | G | - | - | A | A |
| Indel | 31794 | G | - | - | A | A |
| Indel | 31795 | G | - | - | A | A |
| Indel | 31796 | G | - | - | A | A |
| Indel | 31797 | G | - | - | A | A |
| Indel | 31798 | G | - | - | A | A |
| Indel | 31799 | G | - | - | A | A |
| Indel | 31800 | G | - | - | A | A |
| Indel | 31801 | G | - | - | A | A |
| Indel | 31802 | G | - | - | A | A |
| Indel | 31803 | G | - | - | A | A |
| Indel | 31804 | G | - | - | A | A |
| Indel | 31805 | G | - | - | A | A |
| Indel | 31806 | G | - | - | A | A |
| Indel | 31807 | G | - | - | A | A |
| Indel | 31808 | G | - | - | A | A |
| Indel | 31809 | G | - | - | A | A |
| Indel | 31810 | G | - | - | A | A |
| Indel | 31811 | G | - | - | A | A |
| Indel | 31812 | G | - | - | A | A |
| Indel | 31813 | G | - | - | A | A |
| Indel | 31814 | G | - | - | A | A |
| Indel | 31815 | G | - | - | A | A |
| Indel | 31816 | G | - | - | A | A |
| Indel | 31817 | G | - | - | A | A |
| Indel | 31818 | G | - | - | A | A |
| Indel | 31819 | G | - | - | A | A |
| Indel | 31820 | G | - | - | A | A |
| Indel | 31821 | G | - | - | A | A |
| Indel | 31822 | G | - | - | A | A |
| Indel | 31823 | G | - | - | A | A |
| Indel | 31824 | G | - | - | A | A |
| Indel | 31825 | G | - | - | A | A |
| Indel | 31826 | G | - | - | A | A |
| Indel | 31827 | G | - | - | A | A |
| Indel | 31828 | G | - | - | A | A |
| Indel | 31829 | G | - | - | A | A |
| Indel | 31830 | G | - | - | A | A |
| Indel | 31831 | G | - | - | A | A |
| Indel | 31832 | G | - | - | A | A |
| Indel | 31833 | G | - | - | A | A |
| Indel | 31834 | G | - | - | A | A |
| Indel | 31835 | G | - | - | A | A |
| Indel | 31836 | G | - | - | A | A |
| Indel | 31837 | G | - | - | A | A |
| Indel | 31838 | G | - | - | A | A |
| Indel | 31839 | G | - | - | A | A |
| Indel | 31840 | G | - | - | A | A |
| Indel | 31841 | G | - | - | A | A |
| Indel | 31842 | G | - | - | A | A |
| Indel | 31843 | G | - | - | A | A |
| Indel | 31844 | G | - | - | A | A |
| Indel | 31845 | G | - | - | A | A |
| Indel | 31846 | G | - | - | A | A |
| Indel | 31847 | G | - | - | A | A |
| Indel | 31848 | G | - | - | A | A |
| Indel | 31849 | G | - | - | A | A |
| Indel | 31850 | G | - | - | A | A |
| Indel | 31851 | G | - | - | A | A |
| Indel | 31852 | G | - | - | A | A |
| Indel | 31853 | G | - | - | A | A |
| Indel | 31854 | G | - | - | A | A |
| Indel | 31855 | G | - | - | A | A |
| Indel | 31856 | G | - | - | A | A |
| Indel | 31857 | G | - | - | A | A |
| Indel | 31858 | G | - | - | A | A |
| Indel | 31859 | G | - | - | A | A |
| Indel | 31860 | G | - | - | A | A |
| Indel | 31861 | G | - | - | A | A |
| Indel | 31862 | G | - | - | A | A |
| Indel | 31863 | G | - | - | A | A |
| Indel | 31864 | G | - | - | A | A |
| Indel | 31865 | G | - | - | A | A |
| Indel | 31866 | G | - | - | A | A |
| Indel | 31867 | G | - | - | A | A |
| Indel | 31868 | G | - | - | A | A |
| Indel | 31869 | G | - | - | A | A |
| Indel | 31870 | G | - | - | A | A |
| Indel | 31871 | G | - | - | A | A |
| Indel | 31872 | G | - | - | A | A |
| Indel | 31873 | G | - | - | A | A |
| Indel | 31874 | G | - | - | A | A |
| Indel | 31875 | G | - | - | A | A |
| Indel | 31876 | G | - | - | A | A |
| Indel | 31877 | G | - | - | A | A |
| Indel | 31878 | G | - | - | A | A |
| Indel | 31879 | G | - | - | A | A |
| Indel | 31880 | G | - | - | A | A |
| Indel | 31881 | G | - | - | A | A |
| Indel | 31882 | G | - | - | A | A |
| Indel | 31883 | G | - | - | A | A |
| Indel | 31884 | G | - | - | A | A |
| Indel | 31885 | G | - | - | A | A |
| Indel | 31886 | G | - | - | A | A |
| Indel | 31887 | G | - | - | A | A |
| Indel | 31888 | G | - | - | A | A |
| Indel | 31889 | G | - | - | A | A |
| Indel | 31890 | G | - | - | A | A |
| Indel | 31891 | G | - | - | A | A |
| Indel | 31892 | G | - | - | A | A |
| Indel | 31893 | G | - | - | A | A |
| Indel | 31894 | G | - | - | A | A |
| Indel | 31895 | G | - | - | A | A |
| Indel | 31896 | G | - | - | A | A |
| Indel | 31897 | G | - | - | A | A |
| Indel | 31898 | G | - | - | A | A |
| Indel | 31899 | G | - | - | A | A |
| Indel | 31900 | G | - | - | A | A |
| Indel | 31901 | G | - | - | A | A |
| Indel | 31902 | G | - | - | A | A |
| Indel | 31903 | G | - | - | A | A |
| Indel | 31904 | G | - | - | A | A |
| Indel | 31905 | G | - | - | A | A |
| Indel | 31906 | G | - | - | A | A |
| Indel | 31907 | G | - | - | A | A |
| Indel | 31908 | G | - | - | A | A |
| Indel | 31909 | G | - | - | A | A |
| Indel | 31910 | G | - | - | A | A |
| Indel | 31911 | G | - | - | A | A |
| Indel | 32797 | A | - | - | G | G |
| Indel | 33590 | G | - | - | A | A |
| Indel | 33591 | G | - | - | A | A |
| Indel | 33592 | G | - | - | A | A |
| Indel | 33593 | G | - | - | A | A |
| Indel | 34019 | A | - | - | G | G |
| Indel | 34020 | A | - | - | G | G |
| Indel | 34021 | A | - | - | G | G |
| Indel | 34022 | A | - | - | G | G |
| Indel | 34023 | A | - | - | G | G |
| Indel | 34204 | G | - | - | A | A |
| Indel | 34683 | A | - | - | G | G |
| Indel | 34740 | G | - | A | A | A |
| Indel | 34741 | G | A | A | A | A |
| Indel | 34742 | G | A | A | A | A |
| Indel | 34743 | G | A | A | A | A |
| Indel | 34744 | G | A | A | A | A |
| Indel | 34745 | G | A | A | A | A |
| Indel | 34746 | G | A | A | - | - |
| Indel | 34747 | G | A | A | - | - |
| Indel | 34748 | G | A | A | A | A |
| Indel | 34749 | G | A | A | A | A |
| Indel | 34750 | G | A | A | A | A |
| Indel | 34751 | G | A | A | A | A |
| Indel | 34752 | G | A | A | A | A |
| Indel | 34753 | G | A | A | A | A |
| Indel | 34754 | G | A | A | A | A |
| Indel | 34755 | G | A | A | A | A |
| Indel | 34756 | G | A | A | A | A |
| Indel | 34757 | G | A | A | A | A |
| Indel | 34758 | G | A | A | A | A |
| Indel | 34759 | G | A | A | A | A |
| Indel | 34760 | G | A | A | A | A |
| Indel | 34761 | G | A | A | A | A |
| Indel | 35050 | G | - | - | A | A |
| Indel | 35051 | G | - | - | A | A |
| Indel | 35052 | G | - | - | A | A |
| Indel | 35053 | G | - | - | A | A |
| Indel | 35054 | G | - | - | A | A |
| Indel | 35055 | G | - | - | A | A |
| Indel | 35056 | G | - | - | A | A |
| Indel | 35127 | A | G | G | - | - |
| Indel | 35136 | A | - | - | G | - |
| Indel | 35278 | G | - | - | A | A |
| Indel | 35279 | G | - | - | A | A |
| Indel | 35280 | G | - | - | A | A |
| Indel | 35281 | G | - | - | A | A |
| Indel | 35282 | G | - | - | A | A |
| Indel | 35283 | G | - | - | A | A |
| Indel | 35571 | G | - | - | A | A |
| Indel | 36002 | A | - | - | G | G |
| Indel | 36003 | A | - | - | G | - |
| Indel | 36102 | A | - | - | G | G |
| Indel | 36194 | A | - | - | G | G |
| Indel | 36195 | A | - | - | G | G |
| Indel | 36196 | A | - | - | G | G |
| Indel | 36197 | A | - | - | G | G |
| Indel | 36198 | A | - | - | G | G |
| Indel | 36199 | A | - | - | G | G |
| Indel | 36200 | A | - | - | G | G |
| Indel | 36201 | A | - | - | G | G |
| Indel | 36202 | A | - | - | G | G |
| Indel | 36203 | A | - | - | G | G |
| Indel | 36204 | A | - | - | G | G |
| Indel | 36205 | A | - | - | G | G |
| Indel | 36206 | A | - | - | G | G |
| Indel | 36207 | A | - | - | G | G |
| Indel | 36208 | A | - | - | G | G |
| Indel | 36209 | A | - | - | G | G |
| Indel | 36210 | A | - | - | G | G |
| Indel | 36211 | A | - | - | G | G |
| Indel | 36212 | A | - | - | G | G |
| Indel | 36213 | A | - | - | G | G |
| Indel | 36214 | A | - | - | G | G |
| Indel | 36215 | A | - | - | G | G |
| Indel | 36216 | A | - | - | G | G |
| Indel | 36217 | A | - | - | G | G |
| Indel | 36218 | A | - | - | G | G |
| Indel | 36219 | A | - | - | G | G |
| Indel | 36220 | A | - | - | G | G |
| Indel | 36221 | A | - | - | G | G |
| Indel | 36289 | A | G | - | - | - |
| Indel | 36290 | A | G | - | - | - |
| Indel | 36291 | A | G | - | - | - |
| Indel | 36292 | A | G | - | - | - |
| Indel | 36293 | A | G | - | - | - |
| Indel | 36294 | A | G | - | - | - |
| Indel | 36295 | A | G | - | - | - |
| Indel | 36296 | A | G | - | - | - |
| Indel | 36297 | A | G | - | - | - |
| Indel | 36298 | A | G | - | - | - |
| Indel | 36299 | A | G | - | - | - |
| Indel | 36300 | A | G | - | - | - |
| Indel | 36301 | A | G | - | - | - |
| Indel | 36302 | A | G | - | - | - |
| Indel | 36303 | A | G | - | - | - |
| Indel | 36304 | A | G | - | - | - |
| Indel | 36305 | A | G | - | - | - |
| Indel | 36306 | A | G | - | - | - |
| Indel | 36307 | A | G | - | - | - |
| Indel | 36308 | A | G | - | - | - |
| Indel | 36373 | G | - | - | A | A |
| Indel | 36374 | G | - | - | A | A |
| Indel | 36384 | G | A | - | A | A |
| Indel | 36385 | G | A | - | A | A |
| Indel | 36386 | G | A | - | A | A |
| Indel | 36387 | G | A | - | A | A |
| Indel | 36388 | G | A | - | A | A |
| Indel | 36389 | G | A | - | A | A |
| Indel | 36428 | A | - | - | G | G |
| Indel | 36429 | A | - | - | G | G |
| Indel | 36430 | A | - | - | G | G |
| Indel | 36431 | A | - | - | G | G |
| Indel | 39916 | G | A | A | - | - |
| Indel | 39917 | A | - | - | G | G |
| Indel | 40869 | A | - | - | G | G |
| Indel | 40870 | A | - | - | G | G |
| Indel | 40871 | A | - | - | G | G |
| Indel | 40872 | A | - | - | G | G |
| Indel | 40873 | A | - | - | G | G |
| Indel | 40926 | A | - | - | G | G |
| Indel | 41100 | G | - | - | A | A |
| Indel | 41101 | G | - | - | A | A |
| Indel | 41102 | G | - | - | A | A |
| Indel | 41103 | G | - | - | A | A |
| Indel | 41104 | G | - | - | A | A |
| Indel | 41105 | G | - | - | A | A |
| Indel | 41106 | G | - | - | A | A |
| Indel | 41107 | G | - | - | A | A |
| Indel | 41108 | G | - | - | A | A |
| Indel | 41109 | G | - | - | A | A |
| Indel | 41110 | G | - | - | A | A |
| Indel | 41111 | G | - | - | A | A |
| Indel | 41112 | G | - | - | A | A |
| Indel | 41113 | G | - | - | A | A |
| Indel | 41114 | G | - | - | A | A |
| Indel | 41115 | G | - | - | A | A |
| Indel | 41116 | G | - | - | A | A |
| Indel | 41117 | G | - | - | A | A |
| Indel | 41118 | G | - | - | A | A |
| Indel | 41119 | G | - | - | A | A |
| Indel | 41120 | G | - | - | A | A |
| Indel | 41121 | G | - | - | A | A |
| Indel | 41122 | G | - | - | A | A |
| Indel | 41123 | G | - | - | A | A |
| Indel | 41124 | G | - | - | A | A |
| Indel | 41125 | G | - | - | A | A |
| Indel | 41126 | G | - | - | A | A |
| Indel | 41127 | G | - | - | A | A |
| Indel | 41128 | G | - | - | A | A |
| Indel | 41129 | G | - | - | A | A |
| Indel | 41234 | A | G | G | G | G |
| Indel | 41235 | A | G | G | - | - |
| Indel | 47050 | G | - | - | A | A |
| Indel | 49134 | A | - | - | G | G |
| Indel | 49397 | A | - | - | G | G |
| Indel | 49884 | A | - | - | G | G |
| Indel | 50430 | G | A | A | - | - |
| Indel | 50468 | G | - | - | A | A |
| Indel | 50469 | G | - | - | A | A |
| Indel | 50470 | G | - | - | A | A |
| Indel | 50471 | G | - | - | A | A |
| Indel | 50472 | G | - | - | A | A |
| Indel | 50473 | G | - | - | A | A |
| Indel | 50474 | G | - | - | A | A |
| Indel | 50475 | G | - | - | A | A |
| Indel | 50476 | G | - | - | A | A |
| Indel | 50477 | G | - | - | A | A |
| Indel | 50478 | G | - | - | A | A |
| Indel | 50479 | G | - | - | A | A |
| Indel | 50480 | G | - | - | A | A |
| Indel | 50481 | G | - | - | A | A |
| Indel | 50482 | G | - | - | A | A |
| Indel | 50483 | G | - | - | A | A |
| Indel | 50484 | G | - | - | A | A |
| Indel | 50485 | G | - | - | A | A |
| Indel | 50486 | G | - | - | A | A |
| Indel | 50487 | G | - | - | A | A |
| Indel | 50488 | G | - | - | A | A |
| Indel | 51261 | G | - | - | A | A |
| Indel | 52230 | G | - | - | A | A |
| Indel | 53621 | A | - | - | G | G |
| Indel | 53622 | A | - | - | G | G |
| Indel | 53623 | A | - | - | G | G |
| Indel | 53624 | A | - | - | G | G |
| Indel | 53625 | A | - | - | G | G |
| Indel | 53626 | A | - | - | G | G |
| Indel | 53627 | A | - | - | G | G |
| Indel | 53628 | A | - | - | G | G |
| Indel | 53629 | A | - | - | G | G |
| Indel | 53630 | A | - | - | G | G |
| Indel | 53631 | A | - | - | G | G |
| Indel | 53632 | A | - | - | G | G |
| Indel | 53633 | A | - | - | G | G |
| Indel | 53634 | A | - | - | G | G |
| Indel | 53635 | A | - | - | G | G |
| Indel | 53636 | A | - | - | G | G |
| Indel | 53685 | A | - | - | G | G |
| Indel | 53686 | A | - | - | G | G |
| Indel | 53687 | A | - | - | G | G |
| Indel | 53688 | A | - | - | G | G |
| Indel | 54058 | G | - | - | A | A |
| Indel | 54127 | A | G | G | G | G |
| Indel | 54128 | A | G | G | G | G |
| Indel | 54129 | A | G | G | G | G |
| Indel | 54130 | A | G | G | G | G |
| Indel | 54131 | A | G | G | G | G |
| Indel | 54132 | A | G | G | G | G |
| Indel | 54133 | A | G | G | G | G |
| Indel | 54134 | A | G | G | G | G |
| Indel | 54135 | A | G | G | G | G |
| Indel | 54175 | G | - | - | A | A |
| Indel | 54176 | G | - | - | A | A |
| Indel | 54177 | G | - | - | A | A |
| Indel | 54178 | G | - | - | A | A |
| Indel | 54201 | A | - | - | G | G |
| Indel | 54202 | A | - | - | G | G |
| Indel | 54203 | A | - | - | G | G |
| Indel | 55625 | A | G | G | - | - |
| Indel | 56361 | A | - | - | G | G |
| Indel | 56362 | A | - | - | G | G |
| Indel | 56363 | A | - | - | G | G |
| Indel | 56364 | A | - | - | G | G |
| Indel | 56365 | A | - | - | G | G |
| Indel | 56366 | A | - | - | G | G |
| Indel | 56367 | A | - | - | G | G |
| Indel | 56368 | A | - | - | G | G |
| Indel | 56369 | A | - | - | G | G |
| Indel | 56370 | A | - | - | G | G |
| Indel | 56371 | A | - | - | G | G |
| Indel | 57670 | G | - | - | A | A |
| Indel | 57671 | G | - | - | A | A |
| Indel | 57672 | G | - | - | A | A |
| Indel | 57673 | G | - | - | A | A |
| Indel | 57674 | G | - | - | A | A |
| Indel | 57675 | G | - | - | A | A |
| Indel | 57676 | G | - | - | A | A |
| Indel | 57677 | G | - | - | A | A |
| Indel | 57678 | G | - | - | A | A |
| Indel | 57679 | G | - | - | A | A |
| Indel | 57680 | G | - | - | A | A |
| Indel | 57681 | G | - | - | A | A |
| Indel | 57682 | G | - | - | A | A |
| Indel | 57683 | G | - | - | A | A |
| Indel | 57684 | G | - | - | A | A |
| Indel | 57685 | G | - | - | A | A |
| Indel | 57686 | G | - | - | A | A |
| Indel | 57687 | G | - | - | A | A |
| Indel | 57688 | G | - | - | A | A |
| Indel | 57689 | G | - | - | A | A |
| Indel | 57690 | G | - | - | A | A |
| Indel | 57691 | G | - | - | A | A |
| Indel | 57692 | G | - | - | A | A |
| Indel | 57693 | G | - | - | A | A |
| Indel | 57694 | G | - | - | A | A |
| Indel | 57695 | G | - | - | A | A |
| Indel | 64119 | A | - | - | G | G |
| Indel | 64120 | A | - | - | G | G |
| Indel | 64121 | A | - | - | G | G |
| Indel | 64122 | A | - | - | G | G |
| Indel | 64123 | A | - | - | G | G |
| Indel | 64124 | A | - | - | G | G |
| Indel | 64125 | A | - | - | G | G |
| Indel | 64126 | A | - | - | G | G |
| Indel | 64127 | A | - | - | G | G |
| Indel | 64146 | A | - | - | G | G |
| Indel | 64165 | A | - | - | G | G |
| Indel | 64281 | A | G | G | G | G |
| Indel | 64282 | A | - | - | G | G |
| Indel | 64575 | A | G | G | G | G |
| Indel | 64718 | A | - | - | G | G |
| Indel | 64719 | A | - | - | G | G |
| Indel | 64720 | A | - | - | G | G |
| Indel | 64721 | A | - | - | G | G |
| Indel | 64722 | A | - | - | G | G |
| Indel | 64723 | A | - | - | G | G |
| Indel | 64724 | A | - | - | G | G |
| Indel | 64725 | A | - | - | G | G |
| Indel | 64726 | A | - | - | G | G |
| Indel | 64727 | A | - | - | G | G |
| Indel | 64728 | A | - | - | G | G |
| Indel | 64729 | A | - | - | G | G |
| Indel | 64730 | A | - | - | G | G |
| Indel | 65048 | A | G | G | G | G |
| Indel | 65049 | A | - | - | G | G |
| Indel | 65050 | A | - | - | G | G |
| Indel | 65051 | A | - | - | G | G |
| Indel | 65052 | A | - | - | - | G |
| Indel | 66011 | A | G | G | - | - |
| Indel | 66012 | A | G | G | - | - |
| Indel | 66015 | G | - | - | A | A |
| Indel | 66016 | G | - | - | A | A |
| Indel | 68360 | G | - | - | A | A |
| Indel | 68361 | G | - | - | A | A |
| Indel | 68362 | G | - | - | A | A |
| Indel | 68363 | G | - | - | A | A |
| Indel | 68364 | G | - | - | A | A |
| Indel | 68365 | G | - | - | A | A |
| Indel | 68366 | G | - | - | A | A |
| Indel | 68367 | G | - | - | A | A |
| Indel | 68368 | G | - | - | A | A |
| Indel | 68369 | G | - | - | A | A |
| Indel | 68370 | G | - | - | A | A |
| Indel | 68519 | A | G | G | G | G |
| Indel | 68603 | A | - | - | G | G |
| Indel | 68924 | A | G | G | - | - |
| Indel | 69071 | G | - | - | A | A |
| Indel | 70650 | G | - | - | A | A |
| Indel | 70842 | G | - | - | A | A |
| Indel | 70843 | G | - | - | A | A |
| Indel | 70844 | G | - | - | A | A |
| Indel | 70845 | G | - | - | A | A |
| Indel | 70846 | G | - | - | A | A |
| Indel | 70939 | G | - | - | A | A |
| Indel | 71028 | G | - | - | A | A |
| Indel | 71029 | G | - | - | A | A |
| Indel | 71030 | G | - | - | A | A |
| Indel | 71031 | G | - | - | A | A |
| Indel | 71032 | G | - | - | A | A |
| Indel | 71033 | G | - | - | A | A |
| Indel | 71034 | G | - | - | A | A |
| Indel | 71035 | G | - | - | A | A |
| Indel | 71036 | G | - | - | A | A |
| Indel | 71037 | G | - | - | A | A |
| Indel | 71468 | G | - | - | A | A |
| Indel | 71469 | G | - | - | A | A |
| Indel | 71470 | G | - | - | A | A |
| Indel | 71471 | G | - | - | A | A |
| Indel | 71472 | G | - | - | A | A |
| Indel | 71624 | G | - | - | A | A |
| Indel | 71918 | G | - | - | A | A |
| Indel | 71919 | G | - | - | A | A |
| Indel | 72572 | A | G | G | G | G |
| Indel | 72573 | A | - | - | G | G |
| Indel | 72574 | A | - | - | G | G |
| Indel | 73065 | A | - | - | G | G |
| Indel | 73066 | A | - | - | G | G |
| Indel | 75380 | A | G | G | - | - |
| Indel | 76319 | A | G | G | G | G |
| Indel | 76465 | G | - | - | A | A |
| Indel | 77312 | G | - | - | A | A |
| Indel | 77569 | G | - | - | A | A |
| Indel | 77795 | G | A | A | - | - |
| Indel | 78141 | G | - | - | A | A |
| Indel | 78142 | G | - | - | A | A |
| Indel | 78143 | G | - | - | A | A |
| Indel | 78144 | G | - | - | A | A |
| Indel | 78145 | G | - | - | A | A |
| Indel | 78146 | G | - | - | A | A |
| Indel | 78147 | G | - | - | A | A |
| Indel | 78148 | G | - | - | A | A |
| Indel | 78149 | G | - | - | A | A |
| Indel | 78150 | G | - | - | A | A |
| Indel | 78151 | G | - | - | A | A |
| Indel | 78152 | G | - | - | A | A |
| Indel | 78153 | G | - | - | A | A |
| Indel | 78154 | G | - | - | A | A |
| Indel | 78155 | G | - | - | A | A |
| Indel | 78156 | G | - | - | A | A |
| Indel | 78157 | G | - | - | A | A |
| Indel | 80972 | G | A | A | - | - |
| Indel | 81024 | G | - | - | A | A |
| Indel | 81025 | G | - | - | A | A |
| Indel | 81026 | G | - | - | A | A |
| Indel | 81027 | G | - | - | A | A |
| Indel | 81028 | G | - | - | A | A |
| Indel | 81029 | G | - | - | A | A |
| Indel | 81030 | G | - | - | A | A |
| Indel | 81075 | G | - | - | A | A |
| Indel | 81076 | G | - | - | A | A |
| Indel | 81077 | G | - | - | A | A |
| Indel | 81078 | G | - | - | A | A |
| Indel | 81079 | G | - | - | A | A |
| Indel | 81080 | G | - | - | A | A |
| Indel | 81081 | G | - | - | A | A |
| Indel | 81082 | G | - | - | A | A |
| Indel | 81083 | G | - | - | A | A |
| Indel | 81084 | G | - | - | A | A |
| Indel | 81085 | G | - | - | A | A |
| Indel | 81086 | G | - | - | A | A |
| Indel | 81087 | G | - | - | A | A |
| Indel | 81088 | G | - | - | A | A |
| Indel | 81089 | G | - | - | A | A |
| Indel | 81090 | G | - | - | A | A |
| Indel | 81091 | G | - | - | A | A |
| Indel | 81092 | G | - | - | A | A |
| Indel | 81093 | G | - | - | A | A |
| Indel | 81094 | G | - | - | A | A |
| Indel | 81095 | G | - | - | A | A |
| Indel | 81096 | G | - | - | A | A |
| Indel | 81097 | G | - | - | A | A |
| Indel | 81098 | G | - | - | A | A |
| Indel | 81099 | G | - | - | A | A |
| Indel | 81100 | G | - | - | A | A |
| Indel | 81101 | G | - | - | A | A |
| Indel | 81102 | G | - | - | A | A |
| Indel | 81103 | G | - | - | A | A |
| Indel | 81104 | G | - | - | A | A |
| Indel | 81105 | G | - | - | A | A |
| Indel | 81106 | G | - | - | A | A |
| Indel | 81107 | G | - | - | A | A |
| Indel | 81108 | G | - | - | A | A |
| Indel | 81109 | G | - | - | A | A |
| Indel | 81110 | G | - | - | A | A |
| Indel | 81111 | G | - | - | A | A |
| Indel | 81112 | G | - | - | A | A |
| Indel | 81113 | G | - | - | A | A |
| Indel | 81311 | G | A | A | A | A |
| Indel | 81315 | G | - | - | A | A |
| Indel | 82864 | A | - | - | G | G |
| Indel | 82865 | A | - | - | G | G |
| Indel | 82866 | A | - | - | G | G |
| Indel | 82867 | G | A | A | - | - |
| Indel | 83725 | A | G | G | G | G |
| Indel | 83726 | A | - | - | G | G |
| Indel | 85351 | G | - | - | A | A |
| Indel | 85352 | G | - | - | A | A |
| Indel | 85353 | G | - | - | A | A |
| Indel | 85864 | A | G | G | G | G |
| Indel | 85865 | A | - | - | G | G |
| Indel | 85948 | G | A | A | - | - |
| Indel | 86458 | A | G | G | G | G |
| Indel | 86978 | A | - | - | G | G |
| Indel | 87017 | A | - | - | G | G |
| Indel | 87053 | A | - | - | G | G |
| Indel | 87054 | A | - | - | G | G |
| Indel | 87055 | A | - | - | G | G |
| Indel | 87080 | A | - | - | G | G |
| Indel | 90132 | A | - | - | G | G |
| Indel | 104632 | G | - | - | A | A |
| Indel | 104633 | G | - | - | A | A |
| Indel | 104634 | G | - | - | A | A |
| Indel | 104635 | G | - | - | A | A |
| Indel | 104636 | G | - | - | A | A |
| Indel | 104637 | G | - | - | A | A |
| Indel | 104638 | G | - | - | A | A |
| Indel | 104639 | G | - | - | A | A |
| Indel | 104640 | G | - | - | A | A |
| Indel | 104641 | G | - | - | A | A |
| Indel | 104642 | G | - | - | A | A |
| Indel | 104643 | G | - | - | A | A |
| Indel | 116139 | A | - | - | G | G |
| Indel | 116140 | A | - | - | G | G |
| Indel | 116141 | A | - | - | G | G |
| Indel | 116142 | A | - | - | G | G |
| Indel | 116143 | A | - | - | G | G |
| Indel | 116144 | A | - | - | G | G |
| Indel | 116145 | A | - | - | G | G |
| Indel | 118545 | G | A | A | - | - |
| Indel | 118568 | A | - | - | G | G |
| Indel | 118654 | G | - | - | - | A |
| Indel | 118655 | G | - | - | A | A |
| Indel | 118656 | G | - | - | A | A |
| Indel | 118816 | G | - | - | A | A |
| Indel | 118817 | G | - | - | A | A |
| Indel | 118818 | G | - | - | A | A |
| Indel | 118819 | G | - | - | A | A |
| Indel | 118820 | G | - | - | A | - |
| Indel | 118821 | A | G | G | - | - |
| Indel | 118837 | G | - | - | A | A |
| Indel | 119041 | A | - | - | G | G |
| Indel | 119753 | G | - | - | A | A |
| Indel | 119754 | G | - | - | A | A |
| Indel | 120284 | G | - | - | A | A |
| Indel | 120285 | G | - | - | A | A |
| Indel | 120286 | G | - | - | A | A |
| Indel | 120287 | G | - | - | A | A |
| Indel | 120288 | G | - | - | A | A |
| Indel | 120289 | G | - | - | A | A |
| Indel | 121464 | G | - | - | A | - |
| Indel | 121465 | G | - | - | A | - |
| Indel | 121466 | G | - | - | A | - |
| Indel | 121467 | G | - | - | A | - |
| Indel | 121468 | G | - | - | A | - |
| Indel | 121469 | G | - | - | A | - |
| Indel | 121470 | G | - | - | A | - |
| Indel | 121471 | G | - | - | A | - |
| Indel | 121472 | G | - | - | A | - |
| Indel | 121473 | G | - | - | A | - |
| Indel | 121474 | G | - | - | A | - |
| Indel | 121475 | G | - | - | A | - |
| Indel | 121476 | G | - | - | A | - |
| Indel | 121477 | G | - | - | A | - |
| Indel | 121478 | G | - | - | A | - |
| Indel | 121479 | G | - | - | A | - |
| Indel | 121480 | G | - | - | A | - |
| Indel | 121616 | A | - | - | G | G |
| Indel | 124198 | A | - | - | G | G |
| Indel | 124199 | A | - | - | G | G |
| Indel | 124200 | A | - | - | G | G |
| Indel | 124201 | A | - | - | G | G |
| Indel | 124202 | A | - | - | G | G |
| Indel | 124203 | A | - | - | G | G |
| Indel | 124240 | G | - | - | A | A |
| Indel | 124241 | G | - | - | A | A |
| Indel | 124242 | G | - | - | A | A |
| Indel | 124243 | G | - | - | A | A |
| Indel | 124244 | G | - | - | A | A |
| Indel | 126726 | A | G | G | G | G |
| Indel | 126727 | A | G | G | G | G |
| Indel | 126755 | G | - | - | A | A |
| Indel | 126756 | G | - | - | A | A |
| Indel | 126757 | G | - | - | A | A |
| Indel | 126758 | G | - | - | A | A |
| Indel | 126759 | G | - | - | A | A |
| Indel | 126760 | G | - | - | A | A |
| Indel | 126761 | G | - | - | A | A |
| Indel | 126762 | G | - | - | A | A |
| Indel | 126763 | G | - | - | A | A |
| Indel | 126764 | G | - | - | A | A |
| Indel | 126765 | G | - | - | A | A |
| Indel | 126766 | G | - | - | A | A |
| Indel | 126767 | G | - | - | A | A |
| Indel | 126768 | G | - | - | A | A |
| Indel | 126769 | G | - | - | A | A |
| Indel | 126770 | G | - | - | A | A |
| Indel | 126771 | G | - | - | A | A |
| Indel | 126772 | G | - | - | A | A |
| Indel | 126773 | G | - | - | A | A |
| Indel | 126774 | G | - | - | A | A |
| Indel | 126775 | G | - | - | A | A |
| Indel | 126776 | G | - | - | A | A |
| Indel | 126777 | G | - | - | A | A |
| Indel | 126778 | G | - | - | A | A |
| Indel | 126779 | G | - | - | A | A |
| Indel | 126780 | G | - | - | A | A |
| Indel | 126781 | G | - | - | A | A |
| Indel | 126782 | G | - | - | A | A |
| Indel | 126783 | G | - | - | A | A |
| Indel | 126784 | G | - | - | A | A |
| Indel | 126785 | G | - | - | A | A |
| Indel | 126786 | G | - | - | A | A |
| Indel | 126787 | G | - | - | A | A |
| Indel | 126788 | G | - | - | A | A |
| Indel | 126789 | G | - | - | A | A |
| Indel | 126790 | G | - | - | A | A |
| Indel | 126791 | G | - | - | A | A |
| Indel | 126792 | G | - | - | A | A |
| Indel | 126793 | G | - | - | A | A |
| Indel | 126794 | G | - | - | A | A |
| Indel | 126795 | G | - | - | A | A |
| Indel | 126796 | G | - | - | A | A |
| Indel | 126797 | G | - | - | A | A |
| Indel | 126798 | G | - | - | A | A |
| Indel | 126799 | G | - | - | A | A |
| Indel | 126800 | G | - | - | A | A |
| Indel | 126801 | G | - | - | A | A |
| Indel | 126802 | G | - | - | A | A |
| Indel | 126803 | G | - | - | A | A |
| Indel | 126804 | G | - | - | A | A |
| Indel | 126805 | G | - | - | A | A |
| Indel | 126806 | G | - | - | A | A |
| Indel | 126807 | G | - | - | A | A |
| Indel | 126808 | G | - | - | A | A |
| Indel | 126809 | G | - | - | A | A |
| Indel | 126810 | G | - | - | A | A |
| Indel | 127333 | G | A | A | - | - |
| Indel | 127334 | G | A | A | - | - |
| Indel | 129650 | A | - | - | G | G |
| Indel | 129651 | G | A | A | - | - |
| Indel | 130053 | G | - | - | A | A |
| Indel | 132807 | A | - | - | G | G |
| Indel | 132808 | A | - | - | G | G |
| Indel | 132809 | A | - | - | G | G |
| Indel | 132810 | A | - | - | G | G |
| Indel | 132811 | A | - | - | G | G |
| Indel | 132812 | A | - | - | G | G |
| Indel | 132813 | A | - | - | G | G |
| Indel | 132814 | A | - | - | G | G |
| Indel | 132815 | A | - | - | G | G |
| Indel | 133014 | G | - | - | A | A |
| Indel | 133015 | G | - | - | A | A |
| Indel | 133016 | G | - | - | A | A |
| Indel | 133017 | G | - | - | A | A |
| Indel | 133018 | G | - | - | A | A |
| Indel | 133019 | G | - | - | A | A |
| Indel | 133020 | G | - | - | A | A |
| Indel | 133021 | G | - | - | A | A |
| Indel | 133022 | G | - | - | A | A |
| Indel | 146119 | G | - | - | A | A |
| Indel | 146120 | G | - | - | A | A |
| Indel | 146121 | G | - | - | A | A |
| Indel | 146122 | G | - | - | A | A |
| Indel | 146123 | G | - | - | A | A |
| Indel | 146124 | G | - | - | A | A |
| Indel | 146125 | G | - | - | A | A |
| Indel | 146126 | G | - | - | A | A |
| Indel | 146127 | G | - | - | A | A |
| Indel | 146128 | G | - | - | A | A |
| Indel | 146129 | G | - | - | A | A |
| Indel | 146130 | G | - | - | A | A |
| Indel | 160622 | A | - | - | G | G |
